# Supplementary material for: A rapid review to identify physical activity accrued while playing golf
Source: BMJ Open. 2017 Nov 28;7(11):e018993. doi: 10.1136/bmjopen-2017-018993 (PMC5719314; doi:10.1136/bmjopen-2017-018993)
Supplement: Supplementary file 3 [file bmjopen-2017-018993supp003.pdf]

Appendix 3. Sample Data Extraction Form.

Bibliographic Information

**Study ID:**	**Date of extraction:**	**Extracted by:**	**Checked by:**
**Year published:**	**Country:**		
**Title:**			
**Author(s):**			

Study Characteristics

**Aims/purpose:**
**Study design:**
**Analytical methods deployed:**
**Study procedures:**

Participants Characteristics

**Age:**	**Gender:**	**Sample size:**
**Any disease characteristics:**	**Handicap/Average score:**	

Intervention

**Intervention Type:**
**Comparator:**
**Setting:**

**Duration:**	
**Modifiers:**	
Sex	
Hills	
Golf Carts/Pulling/Carrying Clubs	
Age	
Other	

Outcomes

**METS**	
**Energy expenditure**	
**O_2 intake**	
**Heart rate**	
**Steps taken**	
**Distance covered**	
**Strength**	
**Flexibility**	
**Balance**	
**Sedentary Behaviour**	
**Outcome Measures:**	

Results

**Key findings:**
**Limitations:**
