# Supplementary material for: A rapid review to identify physical activity accrued while playing golf
Source: BMJ Open. 2017 Nov 28;7(11):e018993. doi: 10.1136/bmjopen-2017-018993 (PMC5719314; doi:10.1136/bmjopen-2017-018993)
Supplement: Supplementary file 4 [file bmjopen-2017-018993supp004.pdf]

Appendix 4. Results of phase one of the systematic electronic search.

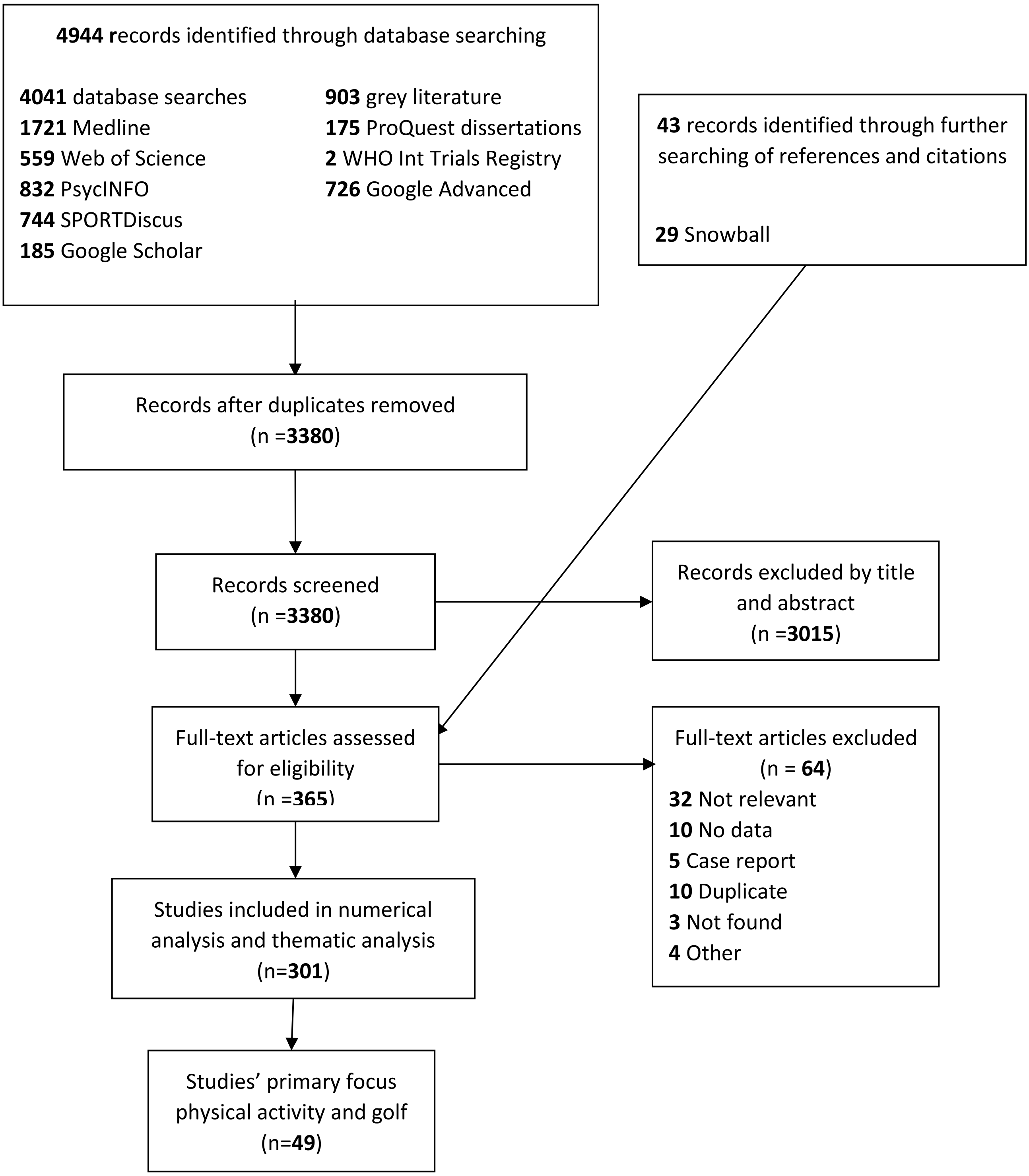

Adapted from Murray et al. [10]
