# Supplementary material for: A rapid review to identify physical activity accrued while playing golf
Source: BMJ Open. 2017 Nov 28;7(11):e018993. doi: 10.1136/bmjopen-2017-018993 (PMC5719314; doi:10.1136/bmjopen-2017-018993)
Supplement: Supplementary file 5 [file bmjopen-2017-018993supp005.pdf]

Appendix 5. Citations of studies included in the rapid review

1. Burkett LN, von Heijne-Fisher U. Heart rate and calorie expenditure of golfers carrying their clubs and walking flat and hilly golf courses. International Sports Journal. 1998;2(2):78-85.

2. Broman G, Johnsson L, Kaijser L. Golf: a high intensity interval activity for elderly men. Aging clinical and experimental research. 2004;16(5):375-81.

3. Crowell BG. Energy cost of participation in golf as determined by telemetry: Oklahoma State University; 1970.

4. Dear JB, Porter MM, Ready AE. Energy expenditure during golfing and lawn mowing in older adult men. J Aging Phys Act. 2010;18(2):185-200.

5. Dobrosielski DA, Brubaker PH, Berry MJ, Ayabe M, Miller HS. The metabolic demand of golf in patients with heart disease and in healthy adults. J Cardiopulm Rehabil. 2002;22(2):96-104.

6. Gabellieri JM. The physiological demands of walking during golf: University of Rhode Island; 2011.

7. Gao KL, Hui-Chan CW, Tsang WW. Golfers have better balance control and confidence than healthy controls. Eur J Appl Physiol. 2011;111(11):2805-12.

8. Kobriger SL, Smith J, Hollman JH, Smith AM. The contribution of golf to daily physical activity recommendations: how many steps does it take to complete a round of golf? Mayo Clin Proc. 2006;81(8):1041-3.

9. Kras J, Larsen B. A comparison of the health benefits of walking and riding during a round of golf. International Sports Journal. 2002;6(1):112-6.

10. Lampley JH, Lampley PM, Howley ET. Caloric cost of playing golf. Res Q. 1977;48(3):637-9.

11. Loy SF. The effect of the game of golf on cardiopulmonary fitness of middle-aged men: California State University, Northridge; 1979.

12. Sell TC, Tsai YS, Smoliga JM, Myers JB, Lephart SM. Strength, flexibility, and balance characteristics of highly proficient golfers. J Strength Cond Res. 2007;21(4):1166-71.

13. Stauch M, Liu Y, Giesler M, Lehmann M. Physical activity level during a round of golf on a hilly course. The Journal of sports medicine and physical fitness. 1999;39(4):321-7.

14. Tsang WW, Hui-Chan CW. Effects of exercise on joint sense and balance in elderly men: Tai Chi versus golf. Medicine and science in sports and exercise. 2004;36(4):658-67.

15. Tsang WW, Hui-Chan CW. Static and dynamic balance control in older golfers. J Aging Phys Act. 2010;18(1):1-13.

16. Unverdorben M, Kolb M, Bauer I, Bauer U, Brune M, Benes K, et al. Cardiovascular load of competitive golf in cardiac patients and healthy controls. Medicine and science in sports and exercise. 2000;32(10):1674-8.

17. Zunzer SC, von Duvillard SP, Tschakert G, Mangus B, Hofmann P. Energy expenditure and sex differences of golf playing. Journal of sports sciences. 2013;31(10):1045-53.

18. Tangen JO, Sunde A, Sageie J, Hagen PC, Kristoffersen B, Istad R, et al. In accordance with governmental recommendations—a study of golf and health. Journal of sports sciences. 2013;1:15-25.

19. Schachten T, Jansen P. The effects of golf training in patients with stroke: a pilot study. Int Psychogeriatr. 2015;27(5):865-73.
