# Supplementary material for: A rapid review to identify physical activity accrued while playing golf
Source: BMJ Open. 2017 Nov 28;7(11):e018993. doi: 10.1136/bmjopen-2017-018993 (PMC5719314; doi:10.1136/bmjopen-2017-018993)
Supplement: Supplementary file 6 [file bmjopen-2017-018993supp006.pdf]

Appendix 6. Characteristics of included studies

Study ID	Author, publication year	Country	Sample size	Participants' age (mean years)	Participants' gender (% Male)	Participants' handicap or average score (mean strokes)	Participant's disease characteristics	Duration	Modifiers	Relevant outcome measures
1	Burkett, 1998	USA	10	24.0	100	<80† (50%)<br>80-95† (50%)	Healthy	3 x 18 holes	Course profile; skill level	Energy expenditure; heart rate
3	Broman, 2004	Sweden	19	27.0 (31%)<br>50.0 (37%)<br>75.0 (31%)	100	NR	1 x Polymyalgia rheumatica; 7 x "back problems"; 2 x knee pain	18 holes	Age; areas of golf course	Heart rate
4	Crowell, 1970	USA	7	42.7	100	11.6	NR	3 x 9 holes	Club transportation; shot type	Energy expenditure; oxygen intake; heart rate; distance covered
7	Dear, 2010	USA	18	71.2	100	NR	Healthy	9 holes	-	METs; energy expenditure; oxygen intake; heart rate; distance covered
8	Dobrosielski, 2002	USA	20	63.6 (50%)<br>65.8 (50%)	100	NR	2 x myocardial infarction<br>4 x coronary artery bypass graft<br>2 x percutaneous coronary intervention<br>1 x cardiomyopathy	9 holes	Heart disease	METs; energy expenditure

11	Gabellieri, 2011	USA	13	28.5	100	97.1†	NR	18 holes	Weight	METs; energy expenditure; heart rate; steps taken; distance covered
12	Gao, 2011	China	23	66.2 (47.8%)<br>71.3 (52.2%)	100	NR	Healthy	NA	-	Balance
24	Kobriger, 2006	USA	42	55	28.6	NR	NR	3 x 18 holes	Sex	Steps taken
25	Kras, 2002	USA	12	42-57	100	7-21	NR	3 x 9 holes	Course profile, club transportation	Heart rate
26	Lampley, 1977	USA	22	34 (50%)<br>32 (50%)	50	19	NR	9 holes	Sex	Energy expenditure
27	Loy, 1979	USA	6	52.5	100	15.7	Healthy	18 holes	-	Energy expenditure; oxygen intake; heart rate
34	Sell, 2008	USA	257	45.5	100	<0 (17.5%)<br>0-9 (46.7%)<br>10-20 (35.8%)	NR	NA	Skill level	Strength; balance; flexibility
35	Stauch, 2003	USA	30	53	70	29	9 x cardiovascular disease	18 holes	Course profile; sex	Heart rate
37	Tsang, 2004	China	35	69.6 (34.3%)<br>66.2 (31.4%)<br>71.3 (34.3%)<br>20.3 (34.3%)	100	NR	NR	NA	-	Balance
38	Tsang, 2010	China	23	66.2 (47.8%)<br>71.3 (52.2%)	100	NR	NR	NA	-	Balance
39	Unverdorben, 2000	Germany	28	65.3 (71.4%)<br>62.0 (28.6%)	100	NR	11 x coronary artery disease; 10 x post myocardial infarction; 2 x	18 holes	Heart disease	METs; oxygen intake; heart rate

							peripheral arterial occlusive disease; 14 x arrhythmias; 1 x post-myocarditis; 2 x valve disease; 1 x diabetes mellitus; 12 x hypertension			
41	Zunzer, 2013	Austria	66	53.3	63.6	26.4 (51.5%)<br>20.4 (48.5%)	NR	9 holes or 18 holes	Sex; course profile; club transportation; age	METs; energy expenditure; heart rate; distance covered
49	Tangen, 2013	Norway	29	44.2	48.3	27.3	NR	18 holes	Sex; course profile; age; skill level	METs; energy expenditure; heart rate; steps taken; distance covered
50	Schachen, 2015	Germany	14	55.1 (50%)<br>53.1 (50%)	NR	NR	Stroke	10 weeks (2 putting session/week)	-	Balance

NR: Not reported
NA: not applicable
**calculated*
†*average score*
