# Supplementary material for: A rapid review to identify physical activity accrued while playing golf
Source: BMJ Open. 2017 Nov 28;7(11):e018993. doi: 10.1136/bmjopen-2017-018993 (PMC5719314; doi:10.1136/bmjopen-2017-018993)
Supplement: Supplementary file 7 [file bmjopen-2017-018993supp007.pdf]

Appendix 7. Quality assessment of included studies.

Author, year	Clear objective	Clearly defined study population	Participation rate of eligible persons >50%	Recruited from same or similar populations + inclusion and exclusion criteria prespecified	Sample size justification, power description, or variance and effect estimates	Exposures measured prior to outcome being measured	Sufficient timeframe	Different levels of exposure examined	Exposure measures clearly defined, valid, reliable and implemented consistently	Exposure assessed more than once over time	Outcome measures clearly defined, valid, reliable and implemented consistently	Outcome assessors blinded to exposure status	Loss to follow-up <20%	Key confounding variables measured and adjusted for	Overall Rating
Burkett, 1998	Y	N	NA	CD	N	NA	Y	Y	N	NA	Y	CD	NA	N	**Fair**
Broman, 2004	Y	Y	NA	Y	N	NA	Y	N	N	NA	Y	NA	NA	N	**Fair**
Crowell, 1970	Y	Y	NA	N	N	NA	Y	Y	Y	NA	Y	CD	NA	Y	**Fair**
Dear, 2010	Y	Y	NA	Y	N	NA	Y	N	N	NA	Y	CD	NA	N	**Fair**
Dobrosielski, 2002	Y	Y	NA	N	N	NA	Y	N	N	NA	Y	CD	NA	N	**Fair**
Gabellieri, 2011	Y	N	NA	CD	Y	NA	Y	N	Y	N	Y	NA	NA	N	**Fair**
Gao, 2011	Y	Y	NA	Y	Y	NA	Y	N	Y	NA	Y	CD	NA	Y	**Good**
Kobriger, 2006	Y	N	NA	Y	Y	NA	Y	Y	N	N	Y	NA	NA	N	**Fair**
Kras, 2002	Y	N	NA	CD	Y	NA	Y	Y	Y	N	Y	CD	NA	N	**Fair**
Lampley, 1977	Y	N	NA	CD	N	NA	Y	N	N	N	Y	NA	NA	Y	**Fair**
Loy, 1979	Y	Y	NA	N	N	NA	Y	N	N	N	Y	NA	NA	N	**Fair**
Sell, 2008	Y	N	NA	CD	N	Y	CD	Y	Y	NA	Y	CD	NA	N	**Good**
Stauch, 2003	Y	Y	NA	N	N	NA	Y	Y	Y	NA	Y	NA	NA	N	**Fair**
Tsang, 2004	Y	N	NA	N	N	Y	Y	N	Y	N	Y	CD	NA	Y	**Fair**

Tsang, 2010	Y	Y	NA	Y	N	Y	Y	N	Y	N	Y	CD	NA	Y	**Good**
Unverdorben, 2000	Y	Y	NA	N	N	NA	Y	N	Y	N	Y	CD	NA	Y	**Good**
Zunzer, 2013	Y	Y	NA	Y	N	NA	Y	Y	Y	N	Y	CD	NA	Y	**Good**
Tangen, 2013	Y	N	NA	CD	N	NA	Y	Y	N	N	Y	CD	NA	Y	**Fair**
Schachen, 2015	Y	N	NA	N	Y	NA	Y	N	Y	NA	Y	CD	NA	N	**Fair**

Y: yes
N: no
NA: not applicable
CD: can't determine
NR: not reported
