# Supplementary material for: A rapid review to identify physical activity accrued while playing golf
Source: BMJ Open. 2017 Nov 28;7(11):e018993. doi: 10.1136/bmjopen-2017-018993 (PMC5719314; doi:10.1136/bmjopen-2017-018993)
Supplement: Supplementary file 1 [file bmjopen-2017-018993supp001.pdf]

Appendix 1. Searching protocol, phase 1

Step 1

SPORT Discus
1st search: from 1900, all articles, all languages
Search for: Golf AND health
Hits: 1364

2nd search
Search for: Golf AND health AND review
Hits: 56, of which 11 relevant studies identified

Google Advanced
1st search: Golf AND health AND review
Hits:487,000
First 200 articles reviewed for relevance, 15 appeared relevant

ProQuest dissertations
1st search: Golf AND health
Hits: 4, of which 2 relevant covering narrow aspect of topic.

Reference section of useful studies reviewed.

Step 2

The main category – terms are:

1. Golf
   Plus/minus
2. Health OR illness OR injur* OR fitness OR mortality OR morbidity

SPORTDiscus

1st search: from 1900, all articles, all languages

Search for: Golf AND health OR illness OR injur* OR fitness OR mortality OR morbidity
Hits: 3781

2nd search: from 1900, all articles excluding magazine, all languages

Search for: Golf AND health OR illness OR injur* OR fitness OR mortality OR morbidity excluding magazines
Hits: 744

Web of Science

1st search: from 1900, all articles, all languages,

Search for: Golf AND health OR illness OR injur* OR fitness OR mortality OR morbidity

Hits: 559

PsycINFO

1st search from 1900, all articles, all languages
Search for: Golf
Topics Hits: 832

Medline

1st search: from 1900, all articles, all languages
Search for: Golf
Hits: 1721

Google scholar

1st search: from 1900, articles and patents, include citations
Search for: Golf AND health OR illness OR injury OR fitness OR mortality OR morbidity
Hits: 154000, >too many

2nd Search: from 1900, articles and patents, include citations
Search for: Golf AND health OR illness OR injury OR fitness OR mortality OR morbidity
Within title
Hits: 185

Initial database search

Hits: 4041 before duplicates
3167 once duplicates removed

Grey Literature

Google (advanced search)

British Journal of Sports Medicine Domain
1st search: from 1900, all articles, all languages
Search for: Golf AND health OR illness OR injury OR fitness OR mortality OR morbidity AND specify URL http://bjsm.bmj.com/
Hits: 548
https://www.google.co.uk/search?as_q=golf&as_epq=&as_oq=health+illness+injury++fitness++mortality+morbidity+&as_eq=&as_nlo=&as_nhi=&lr=&cr=&as_qdr=all&as_sitesearch=http://bjsm.bmj.com/&as_occt=any&safe=images&as_filetype=&as_right=&gws_rd=cr&ei=Eq1AVue-OsfTU6XPtOAC#q=golf+health+OR+illness+OR+injury+OR+fitness+OR+mortality+O R+morbidity+site:http://bjsm.bmj.com/&as_qdr=all&start=20

World Golf Foundation
1st search: from 1900, all articles, all languages
Search for: Golf AND health OR illness OR injury OR fitness OR mortality OR morbidity AND specify URL http://www.worldgolffoundation.org/
Hits:11
https://www.google.com/search?as_q=golf&as_epq=&as_oq=health+illness+injury+fitness+ mortality+morbidity+&as_eq=&as_nlo=&as_nhi=&lr=&cr=&as_qdr=all&as_sitesearch=http %3A%2F%2Fwww.worldgolffoundation.org%2F&as_occt=any&safe=images&as_filetype= &as_rights=

Royal and Ancient
1st search: from 1900, all articles, all languages
Search for: Golf AND health OR illness OR injury OR fitness OR mortality OR morbidity AND specify URL http://www.randa.org/
Hits: 133
https://www.google.com/search?as_q=golf&as_epq=&as_oq=health+illness+injury+fitness+ mortality+morbidity+&as_eq=&as_nlo=&as_nhi=&lr=&cr=&as_qdr=all&as_sitesearch=http %3A%2F%2Fwww.randa.org%2F&as_occt=any&safe=images&as_filetype=&as_rights=

Faculty of Sports and Exercise Medicine
1st search: from 1900, all articles, all languages
Search for: Golf AND health OR illness OR injury OR fitness OR mortality OR morbidity AND specify URL http://www.fsem.ac.uk/
Hits: 8
https://www.google.com/search?as_q=golf&as_epq=&as_oq=health+injury+illness+morbidit y+mortality&as_eq=&as_nlo=&as_nhi=&lr=&cr=&as_qdr=all&as_sitesearch=http%3A%2F %2Fwww.fsem.ac.uk%2F&as_occt=any&safe=images&as_filetype=&as_rights=

American College for Sports Medicine
1st search: from 1900, all articles, all languages
Search for: Golf AND health OR illness OR injury OR fitness OR mortality OR morbidity AND specify URL http://www.acsm.org/
Hits: 26
https://www.google.com/search?as_q=golf&as_epq=&as_oq=health+injury+illness+morbidit y+mortality&as_eq=&as_nlo=&as_nhi=&lr=&cr=&as_qdr=all&as_sitesearch=http%3A%2F %2Fwww.acsm.org%2F&as_occt=any&safe=images&as_filetype=&as_rights=

ProQuest dissertations

1st search: from 1900, all articles, all languages
Search for: Golf AND health OR illness OR injury OR fitness OR mortality OR morbidity

Hits: 42740 >too many

2nd search: Golf AND Health OR illness OR injur* OR fitness OR mortality OR morbidity in title or abstract
Hits: 175 Duplicates 115

World Health Organisation- International Clinical Trials Registry Platform

1st search
Search for: Golf
Hits: 2
http://apps.who.int/trialsearch/Trial2.aspx?TrialID=NCT02544399 (relevant)
http://apps.who.int/trialsearch/Trial2.aspx?TrialID=EUCTR2005-003458-81-IT (not relevant)

Initial Grey Literature Databases search

Hits: 903
