# Supplementary material for: A rapid review to identify physical activity accrued while playing golf
Source: BMJ Open. 2017 Nov 28;7(11):e018993. doi: 10.1136/bmjopen-2017-018993 (PMC5719314; doi:10.1136/bmjopen-2017-018993)
Supplement: Supplementary file 2 [file bmjopen-2017-018993supp002.pdf]

## Appendix 2. Searching protocol, phase 2

**SPORTDiscus**
From 1900, all articles, all languages
Search for: Golf AND health
Hits: 32

**Web of Science**
From 1900, all articles, all languages,
Search for: Golf AND health
Hits: 29

**PsycInfo**
From 1900, all articles, all languages
Search for: Golf
Hits: 10

**Medline**
From 1900, all articles, all languages
Search for: Golf
Hits: 91

**Google Scholar**
From 1900, articles and patents, include citations
Search for: Golf AND health OR illness OR injury OR fitness OR mortality OR morbidity
Within title
Hits: 8
