# Supplementary material for: A rapid review to identify physical activity accrued while playing golf
Source: BMJ Open. 2017 Nov 28;7(11):e018993. doi: 10.1136/bmjopen-2017-018993 (PMC5719314; doi:10.1136/bmjopen-2017-018993)
Supplement: Author's manuscript [file bmjopen-2017-018993.draft_revisions.pdf]

BMJ Open is committed to open peer review. As part of this commitment we make the peer review history of every article we publish publicly available.

When an article is published we post the peer reviewers' comments and the authors' responses online. We also post the versions of the paper that were used during peer review. These are the versions that the peer review comments apply to.

The versions of the paper that follow are the versions that were submitted during the peer review process. They are not the versions of record or the final published versions. They should not be cited or distributed as the published version of this manuscript.

BMJ Open is an open access journal and the full, final, typeset and author-corrected version of record of the manuscript is available on our site with no access controls, subscription charges or pay-per-view fees (http://bmjopen.bmj.com).

If you have any questions on BMJ Open's open peer review process please email editorial.bmjopen@bmj.com

BMJ Open

A rapid review to identify physical activity accrued whilst playing golf

Journal:	*BMJ Open*
Manuscript ID	bmjopen-2017-018993
Article Type:	Research
Date Submitted by the Author:	03-Aug-2017
Complete List of Authors:	Luscombe, Jack; University of Edinburgh, Physical Activity for Health Research Centre; University of Edinburgh, Medical School<br>Murray, Andrew; University of Edinburgh, Physical Activity for Health Research Centre; University of Edinburgh, Sport and Exercise<br>Jenkins, Evan; University of Edinburgh, Physical Activity for Health Research Centre; University of Edinburgh, Medical School<br>Archibald, Daryll; University of Edinburgh, Scottish Collaboration for Public Health Research and Policy
**Primary Subject Heading**:	Public health
Secondary Subject Heading:	Sports and exercise medicine
Keywords:	golf, physical activity, health

SCHOLARONE™
Manuscripts

For peer review only

Title Page

A rapid review to identify physical activity accrued whilst playing golf

Luscombe J[1, 2], Murray AD[2, 3], Jenkins E[1, 2], Archibald D[4]

Corresponding author:

Jack Luscombe, Physical Activity for Health Research Centre, Room 2.33 St Leonard's Land, University of Edinburgh, Edinburgh, UK. EH8 8AQ

Email: s1204551@sms.ed.ac.uk

Telephone: (+44)7772478162

Author affiliations:

[1]Medical School, University of Edinburgh, Edinburgh, United Kingdom.

[2]Physical Activity for Health Research Centre, University of Edinburgh, Edinburgh, United Kingdom.

[3]Sport and Exercise, University of Edinburgh, Edinburgh, United Kingdom.

[4]Scottish Collaboration for Public Health Research and Policy, University of Edinburgh, Edinburgh, United Kingdom.

Keywords: golf, physical activity, health

Word Count: 4214

For peer review only

ABSTRACT

Objective: To identify physical activity accrued whilst playing golf, and modifiers of physical activity accrued.

Design: A rapid review of primary research studies. Quality was assessed using the National Heart, Lung, and Blood Institute quality assessment tool for cohort and cross-sectional studies.

Methods and outcomes: The following databases were searched from 1900 to March 2017: SPORTDiscus, Web of Science, PsycINFO, MEDLINE, Google Scholar, Google Advanced Search, ProQuest, World Health Organisation International Clinical Trials Registry Platform. All primary research investigating golf or golfers with any of the following outcomes was included: metabolic equivalent of task, oxygen uptake, energy expenditure, heart rate, step count, distance covered, strength, flexibility, balance, sedentary behaviour.

Results: Phase one searching identified 4944 citations and phase two searching identified 170 citations. In total 22 articles met inclusion criteria. Golf is primarily a moderate intensity physical activity, but may be low intensity depending upon the playing population and various modifiers. Less physical activity is accrued by those who ride a golf cart compared to those walking the course.

Conclusions: Golf can be encouraged in order to attain physical activity (PA) recommendations. Further research is required into the relationship between golf and strength and flexibility physical activity recommendations, and how modifiers affect physical activity accrued.

Systematic review registration: PROSPERO 2017:CRD42017058237

ARTICLE SUMMARY

Strengths and limitations of this study

- This study is, to our knowledge, the first systematically conducted review to focus on golf and physical activity
- A comprehensive overview of golf and physical activity
- Rapid review – streamlined methods are not subject to the same rigor as a systematic review
- Studies were included regardless of methodological quality, however a sensitivity analysis was performed to evaluate what would happen to the results if studies below a certain established ‘quality threshold’ were systematically excluded

For peer review only - http://bmjopen.bmj.com/site/about/guidelines.xhtml

INTRODUCTION

Physical activity guidelines[1] generally recommend, for adults, at least 150 minutes of moderate intensity activity, or 75 minutes of vigorous physical activity per week, or a combination of the two. In addition, physical activity to improve muscle strength on at least two days a week and efforts to minimise the amount of time spent sedentary are recommended. Moderate intensity physical activity is known to provide longevity, physical and mental health benefits[1-4]. An estimated 41-51% of women and 32-41% of men do not meet these guidelines[5, 6] in the United Kingdom (UK). Furthermore, the proportion of adults meeting guidelines decreases with age - only 7-36% of adults aged 75 and over meet the recommendations[5, 6].

Golf is a popular sport played by over 50 million people[7] of all ages and abilities in over 200 countries[8]. In contrast to the majority of sports, participation is higher in middle-aged and older adults[9-11]. Reviews and guideline documents have suggested golf can provide moderate intensity[1, 12-14] and muscle-strengthening physical activity[13]. These studies have not formally assessed the quality of the evidence.

The frequently-cited Compendium of Physical Activities[11] is a classification of intensity costs of various physical activities. It lists golf as, on average, providing 4.8 metabolic equivalents of task of physical activity, a moderate intensity.

A recently published systematically conducted scoping review[10, 15] provided an overview of golf and health and further highlighted that golf can provide moderate intensity physical activity. As per standard guidelines for undertaking scoping reviews[16], the relative strengths and limitations of included studies were not assessed. There have been no other reviews found that utilise systematic methods exploring physical activity and golf. We therefore aimed to provide a rapid review to identify physical activity accrued whilst playing golf.

Murray et al's[10] scoping review noted several factors that influence the intensity of physical activity whilst playing golf: use of a golf-cart, course profile, age, weight, sex, and baseline fitness of participants[10]. Our secondary aim was therefore to report modifiers to the amount of physical activity accrued whilst playing golf.

METHODS

Our systematic review adhered to our published protocol[17] and followed Preferred Reporting Items for Systematic Reviews and Meta-Analyses (PRISMA) guidelines[18].

Rapid reviews are a streamlined, time efficient and pragmatic approach to synthesise evidence. They have been shown to produce similar conclusions to systematic reviews[19]. Variable methodologies have been described[20], and therefore guidance was sought as to the best approach[21]. A rapid review was conducted due to a short time-frame in which to complete the research. To streamline the process, the search strategy from a recent scoping review[10] was used and adapted, there were less exhaustive searches of grey literature and

For peer review only - http://bmjopen.bmj.com/site/about/guidelines.xhtml

For peer review only

only one reviewer assessed all papers for selection, data extraction and quality assessment compared to a full systematic review.

Search strategy

We adopted a two-phase search strategy. The first phase employed the search strategy used in the recently published scoping review published by team members[10] – a precursor to this rapid review. The scoping review search was undertaken in November 2015 across the following databases: SPORTDiscus, Web of Science, PsycINFO, MEDLINE, Google Scholar, Google Advanced Search, ProQuest dissertations, World Health Organisation International Clinical Trials Registry Platform. The search identified 301 studies relating to the scoping review's aims – the relationship and effects of golf on physical and mental health. Forty-nine of those studies were found to be specifically related to golf and physical activity, which will be used in the current review.

The second phase of the search strategy involved adapting and updating the scoping review search. The search was re-run restricting its scope to search for papers related to golf and physical activity only, that were published from November 2015 to March 2017. A focussed grey literature search was performed using the modified terms 'golf AND health'. The full search strategies can be found in Appendices 1 and 2.

Study selection

One reviewer (JL) selected studies for review first by title and abstract, then by full text review, against inclusion/exclusion criteria with the exception of title and abstract screening of phase two results, conducted by DA. A second reviewer (EJ) independently reviewed a random sample of 10% of the paper by full text review for inclusion or exclusion. Concordance was checked and any discrepancies were discussed and resolved by a team member, either AM or DA.

Inclusion and exclusion criteria were developed through researcher discussion:

Inclusion criteria

- Research articles not limited by geographical location, language or setting
- Research articles published since 1900 up until March 2017
- Research articles discussing any of the following outcomes in relation to golf: metabolic equivalent of task (MET), oxygen uptake, energy expenditure, heart rate, step count, distance covered, strength, flexibility, balance, sedentary behaviour
- Any form of playing golf (including but not limited to 18 holes, nine holes, driving range) or research involving golfers
- All ages groups and both sexes of participants
- Sources of information including randomised control trials, cohort, case-control and cross-sectional studies, that have been synthesised quantitatively

Exclusion criteria

For peer review only - http://bmjopen.bmj.com/site/about/guidelines.xhtml

- Studies focussing exclusively on caddies and/or spectators
- Qualitative studies, reviews, opinion pieces, magazine and newspaper articles, case reports, conference proceedings.

Data extraction

Data was extracted by one reviewer (JL) using a data extraction form. The data extraction form was piloted using 10% of papers and modifications were made. A random sample of 10% was independently extracted by a second reviewer (EJ) and results compared. Concordance was checked and any discrepancies were discussed and resolved by a team member, either AM or DA. A sample data extraction form can be found in Appendix 3.

Quality assessment

Our protocol[17] details use of the Effective Public Health Practice Project's quality assessment tool for quantitative studies[22] to assess study quality. After trialling, it became apparent the tool was more suited to interventional studies with groups. As the large majority of included studies are observation cross-sectional design, the tool was not suitable and therefore the National Heart, Lung, and Blood Institute quality assessment tool for observational cohort and cross-sectional studies[23] was used. Eligible studies were assessed by one reviewer (JL). A second reviewer (EJ) independently assessed a random sample of 10% of the papers using the same tool. Concordance was checked and any discrepancies were discussed and resolved by a third researcher, either AM or DA. Studies were included regardless of their methodological quality due to the limited available evidence. It was felt that studies rated 'Poor' could add potentially interesting insights. Sensitivity analysis was conducted to determine whether exclusion of 'Poor' quality studies would have altered results.

Data synthesis and analysis

Due to the wide heterogeneity of included studies in terms of study design, population, setting, outcomes and study quality, data were synthesised narratively with summary tables and figures using the following outcomes: metabolic equivalent of task (MET), energy expenditure, oxygen uptake, heart rate, steps taken, distance covered, strength, flexibility, balance and sedentary behaviour. Modifiers to physical activity accrued were noted during data extraction and were also narratively synthesised. There were no principal summary measures due to the studies' heterogeneity; data were presented using the raw outcome measures.

RESULTS

Study Selection

In total, 3550 independent records were identified through our systematic two-phase electronic search. 3380 independent records were identified in phase one[10]. 3015 records were excluded on screening of title and abstract, and 64 articles were excluded by full text review. Of the remaining 301 articles, 49 were

For peer review only - http://bmjopen.bmj.com/site/about/guidelines.xhtml

specifically related to golf and physical activity. A flowchart detailing the results of phase one of the search can be found in Appendix 4.

Phase two of the search identified 170 further independent records (Figure 1). 168 records were excluded by title and abstract. The 49 articles from phase one were included here and assessed for eligibility by full text review. 29 articles were excluded by full text review. 22 articles remained that met the inclusion criteria and were included in the review. Citations of included studies can be found in Appendix 5.

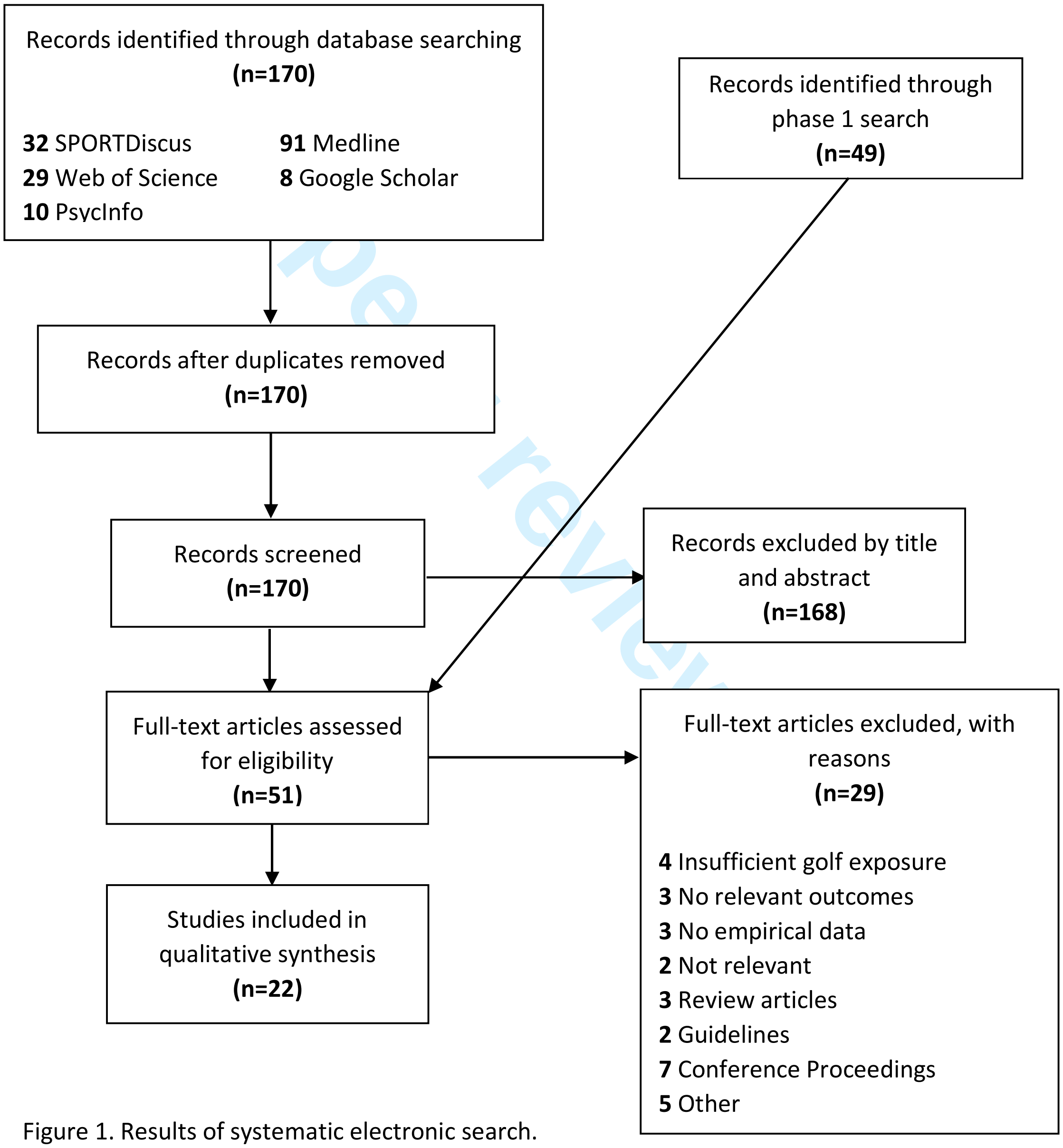

Figure 1. Results of systematic electronic search.

Study Characteristics

For peer review only - http://bmjopen.bmj.com/site/about/guidelines.xhtml

Of the 22 included studies, 12 were conducted in United States, 3 in China and 7 in other countries (Germany, Sweden, New Zealand, Japan, Austria and Norway). 21 of the studies were cross sectional design and 1 study was cohort design. 18 of the identified studies were primary published research papers, 4 were published dissertations. The studies' publication dates range from 1965 – 2015. 6 of the studies were published pre-2000, 16 studies were published post-2000.

10 different outcome measures were used in the review. The most frequently reported were: heart rate (12 studies), energy expenditure (10 studies) and METs (7 studies). No studies reported on sedentary behaviour. Further characteristics of included studies are presented in Appendix 6.

Quality of Included Studies

Information on quality assessment of included studies can be found in Appendix 7. All studies provided a clear objective or research question. Most studies (73%) did not provide a sample size justification, power description or variance/effect estimates. It was unclear in the majority of studies whether outcome assessors were blinded to exposure status of participants (64%). Five studies were rated 'Good', 14 were rated 'Fair' and 3 were rated 'Poor'.

Outcomes

Energy Expenditure

10 studies identified energy expenditure (EE) as an outcome[24-33]. 8 studies were rated 'Fair'[24-30, 33], 1 'Good'[32] and 1 'Poor'[31]. Results are detailed in Table 1. Two studies found significantly higher energy expenditure on hillier courses compared to flatter courses. Zunzer et al.[32], however, found no significant difference in energy expenditure between hilly and flat courses.

Lampley et al.[29] noted a significantly higher rate of energy expenditure in women. In contrast, two studies[32, 33] found males expended significantly more energy than females. However, Zunzer et al.[32] notes that this is not significant if body mass is accounted for and Tangen et al.[33] suggests this may be due to differences in course distance.

Two studies[24, 33] found no significant difference in energy expenditure in relation to skill level.

Crowell[25] notes the lowest energy expenditure when riding a golf cart, then pulling clubs and highest when carrying clubs. Zunzer et al.[32] found those who rode a golf cart had significantly lower energy expenditure than those who pulled or carried clubs. Tangen et al.[33] found no significant difference in relation to club transportation; however it is noted that this may be due to small sample size in each group.

For peer review only - http://bmjopen.bmj.com/site/about/guidelines.xhtml

For peer review only

Study	Quality Assessment	No. of Holes	Club Transportation	Course Profile	EE *(kcal·min^{-1})*	Net EE *(kcal)*	Gross EE *(kcal)*	EE *(kcal·kg·hr^{-1})*
Burkett et al.[24]	Fair	18	Carrying clubs	Flat<br>Medium<br>Hilly	7.25 ± 1.75<br>8.15 ± 1.79<br>8.25 ± 1.83	-	-	-
Crowell[25]	Fair	9	Riding a golf cart<br>Pulling clubs<br>Carrying clubs	*Not reported*	5.2<br>6.8<br>7.5	-	-	-
Dear et al.[26]	Fair	9	Pulling clubs	*Not reported*	-	310.3 ± 83.9	511.6 ± 115.5	-
Dobrosielski et al.[27]	Fair	9	Pulling clubs	Mixed	-	458	-	--
Gabellieri[28]	Fair	18	Carrying clubs	"Undulating"	-	-	1202.8 ± 465.2	
Lampley et al.[29]	Fair	9	Pulling clubs	*Not reported*	-	-	-	4.2 ± 0.6 *(male)*<br>4.8 ± 0.4 *(female)*
Loy[30]	Fair	18	Carrying clubs	Hilly	6.2 ± 0.6	-	-	4.8*
Murase et al.[31]	Poor	18	*Not reported*	Medium	5.9 ± 0.9	-	-	-
Zunzer et al.[32]	Good	9<br><br><br>18	Mixed	Mixed	-	-	520 ± 133 *(male)*<br>273 ± 66 *(female)*<br>926 ± 292 *(male)*<br>556 ± 180 *(female)*	-
Tangen et al.[33]	Fair	18	Mixed	Hilly	-	-	2467 *(male)*<br>1587 *(female)*	-

Table 1. Energy expenditure for a round of golf.

*calculated for a 68kg man

For peer review only - http://bmjopen.bmj.com/site/about/guidelines.xhtml

Metabolic Equivalent of Task

Seven studies identified metabolic equivalent of task (MET) as an outcome[26-28, 32-35]. Four of the studies were rated 'Fair'[26-28, 33], 2 'Good'[32, 35] and 1 'Poor'[34]. Results are detailed in Table 2. Dobrosielski et al.[27] found a significant difference between cardiac patients and healthy adults in average MET (57 ± 2.7; 46 ± 2.6% peak MET) and peak MET (89 ± 3.3; 77 ± 3.6% peak MET). However, Unverdorben et al.[35] found the same MET value (3.1) for cardiac patients and healthy adults. Zunzer et al.[32] notes no significant difference in METs between sexes; whereas Tangen et al.[33] found an almost significant difference between men and women (p=0.069). Zunzer et al.[32] found no significant difference in METs between hilly and flat golf courses.

**Study**	**Quality Assessment**	**No. of Holes**	**Club Transportation**	**METs** *(mean ± SD)*
Dear et al.[26]	Fair	9	Pulling clubs	2.8 ± 0.5
Dobrosielski et al.[27]	Fair	9	Pulling clubs	4.1 ± 0.1 *(cardiac disease)*
Gabellieri[28]	Fair	18	Carrying clubs	8.6 ± 3.1
Moy et al.[34]	Poor	*Not reported*	*Not reported*	5.3
Unverdorben et al.[35]	Good	18<br>18	Pulling clubs<br>Pulling clubs	3.1 *(cardiac disease)*<br>3.1 *(controls)*
Zunzer et al.[32]	Good	9<br>18	Mixed<br>Mixed	2.9 ± 0.8 *(male)*<br>2.2 ± 0.6 *(female)*<br>2.8 ± 0.7 *(male)*<br>2.1 ± 0.7 *(female)*
Tangen et al.[33]	Fair	18	Mixed	5.8 *(male)*<br>4.9 *(female)*

Table 2. Metabolic equivalent of task of a round of golf.

Heart Rate

12 studies reported heart rate (HR) as an outcome[24, 25, 28, 30-33, 35-39]. 8 were rated 'Fair'[24, 25, 28, 30, 33, 36, 38, 39], 2 'Good'[32, 35] and 2 'Poor'[31, 37]. Mean HR and mean percentage of maximum HR (%HR_{max}) are presented in Table 3. In relation to maximum HR, Stauch et al.[36] found most time during a round of golf is spent at 50-74%HR_{max}. Tangen et al.[33] describes 75% of a golf round is at <70%HR_{max} and 25% is >70%. Broman et al.[36] found 70% of total time for elderly men is at >70%HR_{max}; whereas, for middle-aged and younger men, most time is spent at <70%HR_{max}. Loy[30] estimates 75.25 minutes are >60% heart rate reserve.

Getchell[37] studied the effects of a season of golf on various measures of HR. He found no significant difference in resting HR, but found significant decreases in HR towards the end of a submaximal treadmill test and during recovery as well as a significant difference in HR between golfers and controls towards the end of the test.

For peer review only

One paper[24] notes a significant difference in mean heart rate and a second paper[38], time spent >40%HR_{max} between hillier and flatter courses. Two papers found no significant difference in mean HR in relation to course profile[32, 33]. However, Tangen et al.[33] found a significantly higher maximum HR on the hillier course.

Two papers found highest heart rates when carrying clubs, then pulling clubs and lowest when riding a golf cart[25, 38]. One of these studies[38] found a significant difference in percentage of time spent >40%HR_{max} between carrying and pulling clubs, and riding a golf cart. Similarly, Zunzer et al.[32] found participants who rode a golf cart had significantly lower mean HR than those who carried or pulled their clubs. Stauch et al.[39] observed no significant difference in mean or maximum HR in relation to club transportation. However, it is noted there are significant differences in ages, a possible modifier to physical activity attained, between groups – this was also observed in another study[32].

Crowell[25] notes little difference in mean HR in relation to skill level and Burkett et al.[24] found no significant difference. In relation to sex, two papers[32, 33] observed no significant difference in mean HR and one paper[32], minimum, maximum HR or mean percentage HR_{max}. Broman et al.[36] found older golfers spent significantly more time at higher %HR_{max} than middle-aged or younger golfers. Tangen et al.[33] found older golfers (>50yrs) spent less time at high intensity level (>120bpm) than younger golfers (<50yrs) – but suggests this may be due to differences in maximum HR. Unverdorben et al.[35] observed no significant difference in mean HR between cardiac patients and healthy controls; but notes the maximum HR of controls was higher and therefore cardiac patients may work harder.

For peer review only - http://bmjopen.bmj.com/site/about/guidelines.xhtml

Study	Quality Assessment	No. of Holes	Club transportation	Course Profile	Mean HR (bpm)	Mean %HRmax
Burkett et al.[24]	Fair	18	Carrying clubs	Flat<br><br>Medium<br><br>Hilly	108.20 ± 13.16 *(GS)*<br>110.80 ± 7.26 *(AS)*<br>121.80 ± 18.54 *(GS)*<br>117.80 ± 13.54 *(AS)*<br>123.80 ± 21.81 *(GS)*<br>116.20 ± 14.97 *(AS)*	-
Crowell[25]	Fair	9	Riding a golf cart<br>Pulling clubs<br>Carrying clubs	*Not reported*	89.1 ± 10.6<br>103 ± 9.2<br>113.1 ± 8.8	-
Gabellieri[28]	Fair	18	Carrying clubs	"Undulating"	103.5 ± 13.2	55.2 ± 7.4
Loy[30]	Fair	18	Carrying clubs	Hilly	124.7 ± 8.6	
Murase et al.[31]	Poor	18	*Not reported*	Medium	108 ± 10.9	-
Stauch et al.[39]	Fair	18	Riding a golf cart<br>Pulling clubs<br>Carrying clubs	Hilly	111.0 ± 14.0<br>107.2 ± 11.0<br>118.4 ± 17.0	-
Unverdorben et al.[35]	Good	18	Pulling clubs	Hilly	105.4 ± 10.6 *(cardiac patients)*<br>100.5 ± 7.3 *(controls)*	-
Zunzer et al.[32]	Good	9<br><br>18	Mixed	Mixed	101 ± 12 *(male)*<br>99 ± 13 *(female)*<br>105 ± 14 *(male)*<br>103 ± 12 *(female)*	59.2 ± 3.1 *(male)*<br>59.2 ± 8.9 *(female)*<br>60.9 ± 8.6 *(male)*<br>61.6 ± 7.7 *(female)*
Tangen et al.[33]	Fair	18	Mixed	Hilly	104.1 ± 14.5 *(male)*<br>110.8 ± 16.9 *(female)*	-

Table 3. Mean heart rate and percentage of maximum heart rate during a round of golf.

GS: good skill (score<80)

AS: average skill (score 80-95)

For peer review only

Oxygen Uptake

Six studies listed oxygen uptake as an outcome[25, 26, 30, 31, 35, 37]. 3 were rated 'Fair'[25, 26, 30], 1 'Poor'[31] and 1 'Good'[35]. Results are detailed in Table 4. Crowell[25] found riding a golf cart required least oxygen uptake per minute, then pulling clubs, and carrying clubs required the most oxygen uptake per minute. The study also noted golfers of lower handicaps (≤10) required less oxygen per minute when pulling or carrying clubs than golfers with higher handicaps (≥11). Dear et al.'s[26] value of 9.9 ± 1.7ml·kg^{-1}·min^{-1} equates to 34.4 ± 9.1% oxygen uptake reserve. Unverdorben et al.[35] found cardiac patients had a significantly higher %VO_2max whilst playing golf compared to healthy controls.

Getchell[37] found that, after a season of golf, there was no significant difference within or between groups (golfer and controls) in resting oxygen uptake. There was a significant decrease in oxygen uptake for the first 3 minutes and 3rd-10th minute recovery following a treadmill test. However, controls also showed a significant decrease in oxygen uptake for the 3rd-10th minute recovery.

**Study**	**Quality Assessment**	**No. of Holes**	**Club Transportation**	**Oxygen Uptake (l·min^{-1})** *(mean ± SD)*	**Oxygen Uptake (ml·kg^{-1}·min^{-1})** *(mean ± SD)*	**%VO2max**
Crowell[25]	Fair	9<br>9<br>9	Riding a golf cart<br>Pulling clubs<br>Carrying clubs	1.05 ± 0.11<br>1.37 ± 0.03<br>1.50 ± 0.11	8.5<br>9.1<br>9.7	-
Dear et al. [26]	Fair	9	Pulling clubs	-	9.9 ± 1.7	-
Loy[30]	Fair	18	Carrying clubs	1.23 ± 0.11	-	
Murase et al.[31]	Poor	18	Not reported	1.17 ± 0.18	-	38.2 ± 2.1
Unverdorben et al.[35]	Good	18<br><br>18	Pulling clubs	-	-	76.0 ± 13.1 *(cardiac patients)*<br>55.3 ± 9.1 *(controls)*

Table 4. Oxygen uptake during a round of golf.

Steps Taken

3 articles were found with steps taken as an outcome[28, 33, 40]. All studies rated 'Fair' in quality assessment. The included studies all involved an 18-hole round of golf. Studies found 11245±1351[28], 11948±1781[40], 16080±1195 (male)[33] and 16667±992 (female)[33] steps were taken during a round of golf. One study[28] found significant negative correlation between number of steps taken and: weight of the golf bag ($p<0.05$), energy expenditure ($p<0.01$) and minimum heart rate ($p<0.01$) of participants.

Distance Covered

For peer review only - http://bmjopen.bmj.com/site/about/guidelines.xhtml

5 studies detailed distance covered as an outcome[25, 26, 28, 32, 33]. 4 of the studies were rated 'Fair'[25, 26, 28, 33] and 1 study[33] was rated 'Good'. Results are detailed in Table 5. With the exception of Crowell[25], all studies estimated between 8.7 and 11.25km walked for an 18-hole course, and 4.4 and 5.32km for a 9-hole course. Distance covered is highly dependent on the individual golf course length. The course in Crowell's study is poorly described, but this may account for the shorter distance. A much shorter distance (3.18km) is walked riding a golf cart compared to pulling a golf cart or carrying clubs[25]. There is no notable difference in distance walked when pulling a golf cart compared to carrying clubs. Males walked longer distances than females. Zunzer et al.[32] noted a significant difference between male and female distance walked over 18 holes. However, in both studies[32, 33] and as is usual on golf courses, the men's course is longer than the women's. Tangen et al.[33] found that, when course length is accounted for, women (2.13x course length) walked significantly longer than men (1.98x course length).

**Study**	**Quality Assessment**	**No. of holes**	**Club Transportation**	**Sex**	**Distance (km, mean ± SD)**
Crowell[25]	Fair	18	Riding a golf cart<br>Pull cart<br>Carrying clubs	Male	3.18 ± 0.56*<br>7.37 ± 0.71*<br>6.47 ± 0.84*
Dear et al.[26]	Fair	9	Pull cart	Male	4.4 ± 3.6
Gabellieri[28]	Fair	18	Carrying clubs	Male	8.7 ± 0.6*
Zunzer et al.[32]	Good	18<br><br>9	Mixed<br>Mixed<br>Mixed<br>Mixed	Male<br>Female<br>Male<br>Female	10.54 ± 0.94<br>9.89 ± 0.81<br>5.32 ± 0.48<br>5.25 ± 0.76
Tangen et al.[33]	Fair	18	Mixed<br>Mixed	Male<br>Female	11.25 ± 0.83<br>10.00 ± 0.56

*converted to kilometres

Table 5. Distance covered in a round of golf.

Strength

Two studies listed strength as an outcome[37, 41]. One study[37] rated 'Poor', the other[41] rated 'Good'. Getchell[37] found no significant differences in cable strength tension tests, chins or dips within or between groups (golfers and controls), before and after a season of golf. A significant decrease was observed in right and left hand grip strength in golfers compared to healthy controls. There was a significant difference in total dynamometer strength and total dynamometer strength adjusted for body weight, between golfers and controls. Information on the amount of golf played during the season was extremely limited, and therefore findings should be interpreted with caution. Sell et al.[41] found golfers with a lower handicap (<0) had significantly greater strength over a range of measures when compared to handicaps of 0-9 and 10-20. Tables are not listed for strength, flexibility or balance outcomes due to the heterogeneity of measurements.

For peer review only - http://bmjopen.bmj.com/site/about/guidelines.xhtml

Flexibility

Two studies listed flexibility as an outcome[37, 41]. One study[37] rated 'Poor', the other[41] rated 'Good'. Getchell[37] found no significant differences within or between groups (golfers and controls) in trunk forward flexibility and back hyper-extension, before and after a season of golf. Sell et al.[41] found golfers with a lower handicap (<0) had significantly greater range of motion in several measures of shoulder, hip, torso flexibility than golfers with higher handicaps (0-9 and 10-20).

Balance

Five studies listed balance as an outcome[41-45]. Three studies rated 'Good'[41, 42, 44], two studies rated 'Fair'[43, 45]. Three studies focussed on older golfers[42-44] and all papers found elderly golfers had significantly better balance control when compared to controls over a variety of measures. Tsang et al.[43] notes that the balance of elderly golfers was comparable to that of young controls (no significant difference).

Sell et al.[41] found golfers with better handicaps (<0) had significantly better single-leg balance than golfers with handicaps 0-9 and 10-20. Schachten et al.[45] noted a significant improvement in stroke patients after participating in a 10 week, 20 session golf putting intervention. However, a significant improvement was also noted in the comparator group and no significant difference was observed between groups.

Sensitivity analysis

As stated above, we opted not to exclude studies rated as methodologically poor during the quality assessment stage of the review. This decision was taken as there were relatively few studies eligible for inclusion and it was felt that low-rated studies may potentially offer valuable insights in regards to the review question. Nevertheless, it was also felt that it would be important to evaluate how these low quality studies impacted on the findings by undertaking a brief sensitivity analysis[46]. This analysis considered how the results of the study would be affected if the low quality studies were excluded.

Of the three 'Poor' studies, two papers[31, 34] produced data that corresponds with other included studies. Moy et al.[34] found golf equated to 5.3 METs – this value is well within the range of other included studies (2.1 – 8.6 METs). Murase et al.[31] produced values of 1.17 $l\cdot min^{-1}$ oxygen uptake, 5.9$kcal\cdot min^{-1}$ mean energy expenditure and 108bpm mean heart rate. These values are also within ranges of other included studies for oxygen uptake (1.05 - 1.50 $l\cdot min^{-1}$), energy expenditure (5.2 – 8.25$kcal\cdot min^{-1}$) and heart rate (89.1 – 124.7bpm). The exclusion of these studies is therefore unlikely to have significantly affected the results.

The third study[37] is the only cohort study included. It studies the effect of a season of golf on several outcome measures and finds little difference in many of them. This paper is unique in the included studies. It found several non-significant differences in oxygen uptake, heart rate, strength and flexibility measures between golfers and controls, and before and after a season of golf. The conclusions of this study broadly differ from the majority of studies and therefore excluding this study may have altered the results, particularly in regards to the following outcomes. There were no

For peer review only - http://bmjopen.bmj.com/site/about/guidelines.xhtml

significant differences in resting HR, but significant decreases in HR during a submaximal treadmill test and during recovery, as well as a significant difference between golfers and controls. Resting oxygen uptake and oxygen uptake during a treadmill test was not significantly different between or within groups, but golfers controls both exhibited a significant decrease in oxygen uptake during recovery. There were no significant differences between or within groups in cable strength tension tests, chins, dips, trunk forward flexibility and back hyper-extension. A significant difference was noted in total dynamometer strength and when adjusted for body weight between golfers and controls. Notably, there was a significant decrease in right and left hand grip strength in golfers compared to controls.

Whilst the large majority of the results from this study are outliers, it has provided unique methods and interesting insights that diverge from other included studies.

DISCUSSION

Energy expenditure for an 18-hole round of golf appears to achieve the America College of Sports Medicine's (ACSM) recommendation of $1000kcal \cdot wk^{-1}$ [47], and could be separated into two 9-hole rounds. The length of time a round of golf takes can compensate for the low energy expenditure per minute. 57% of MET values stated are within the range of moderate intensity (3-5.9)[47]. Values for $\%HR_{max}$ are within light intensity (50-63%) and moderate intensity (64-76%)[14]. Using the mean age of golfers in UK (63yrs[48]), the mean range for moderate intensity is 101-119bpm – the large majority of data fall into this category.

There were varied results in oxygen uptake. In terms of VO_2max, studies classified golf as light (37-45% VO_2max), moderate (46-63% VO_2max) and vigorous (64-90%VO_2max)[47]. Many studies were close to, but did not reach, the moderate intensity threshold of $10.5\text{-}20.7ml \cdot kg^{-1} \cdot min^{-1}$ (3-5.9 METs), and would therefore be classified as light activity ($<10.5ml \cdot kg^{-1} \cdot min^{-1}$).

All included studies, on average, attained the often cited 10,000 steps[47] during an 18-hole round and, according to Tudor-Locke et al.[49], would be classed as moderate-to-vigorous physical activity. Distance walked is highly variable depending on the course; values range from 6.4-11.3km for an 18-hole round and 4.4-5.3km for a 9-hole round. In relation to strength, flexibility and balance, greater strength and range of motion was found in those with higher proficiency[41]. It is unclear whether this is due to increased volume of play, additional strength/flexibility work or whether these characteristics are likely to lead to a lower handicap. Furthermore, there appears to be better balance control in golfers suggesting that the complex motion while swinging a club and/or walking on uneven grounds during golf play may lead to improved stability.

Evidence suggests use of an electric golf cart significantly reduces physical activity attained in terms of energy expenditure, heart rate and distance covered. Males expend more energy and walk further distances than females. However, it is likely that this due to increased body mass and greater course length played by males. When course length is accounted for, women walk significantly longer[33]. Skill level does not appear to affect physical activity accrued, with the possible exception of

For peer review only - http://bmjopen.bmj.com/site/about/guidelines.xhtml

strength, balance and flexibility. The evidence is unclear whether course profile and age affect physical activity accrued.

This study is, to our knowledge, the first systematically conducted review to focus exclusively on golf and physical activity. It provides a general overview of physical activity accrued whilst playing golf. A rapid review was conducted due to time constraints. Rapid reviews make use streamlined methods and, due to this, are not subject to the same rigor as systematic reviews. Studies were included regardless of their methodological quality. Due to this, three of the included studies were of poor quality. For some outcomes, there was little available evidence.

In agreement with the recent scoping review[10] and the Compendium of Physical Activities[11], golf can provide moderate intensity physical activity. Exercise intensity varies during the game itself. For certain populations, it may be primarily a low intensity physical activity. Shortfalls in intensity, however, are compensated for by the length of the game. Therefore, golf is a viable sport by which to achieve the physical activity recommendations[1]. Golfers may find it difficult to play enough during a week in order to reach PA recommendations, and may wish to supplement golf with another physical activity. Clinicians and policymakers can be encouraged to suggest golf as a form of physical activity in order to meet recommended levels and attain health benefits.

Further research is warranted to investigate whether strength and flexibility is accrued whilst playing golf as well as research examining the effect of modifiers such as age, course profile, disease characteristics and carrying or pulling clubs, on physical activity attained.

CONCLUSION

This rapid review identified 22 articles that examined golf and physical activity. Golf is primarily a moderate intensity physical activity, but may be low intensity or even high intensity depending on the population and various modifiers present. If able, golfers should walk the course, pulling or carrying clubs, rather than ride a golf cart to maximise health benefits. Course profile, skill level and age may affect the amount of physical activity accrued, further research is required.

DECLARATION OF COMPETING INTERESTS

Although not for this project, AM has previously received funding to complete research from the World Golf Foundation. The World Golf Foundation committed to publishing whether results were positive, negative or equivocal, and had no influence on the conduct of this, or previous research.

FUNDING STATEMENT

This work was supported by the Medical Research Council (MRC; MR/K023209/1).

AUTHORS' CONTRIBUTIONS

Jack Luscombe: Lead researcher. Performed study selection, data extraction, quality assessment and narrative synthesis.

For peer review only - http://bmjopen.bmj.com/site/about/guidelines.xhtml

Dr Andrew Murray: Primary supervisor. Provided advice on methods including study selection criteria, data extraction and presentation of data. Performed phase one of the search strategy (previously published).

Evan Jenkins: Independently reviewed 10% of paper for study selection, data extraction and quality assessment.

Dr Daryll Archibald: Senior researcher. Performed phase two of search strategy and phase two study selection by title and abstract. Provided advice on methods including study selection criteria, data extraction and quality assessment.

REFERENCES

1. Department of Health PA, Health Improvement and Protection. Start active, stay active: A report on physical activity from the four home countries' Chief Medical Officers. 2011.
2. Reid H, Foster C. Infographic. Physical activity benefits for adults and older adults. British journal of sports medicine. 2016.
3. Warburton DE, Charlesworth S, Ivey A, Nettlefold L, Bredin SS. A systematic review of the evidence for Canada's Physical Activity Guidelines for Adults. The international journal of behavioral nutrition and physical activity. 2010;7:39.
4. O'Donovan G, Blazevich AJ, Boreham C, Cooper AR, Crank H, Ekelund U, et al. The ABC of Physical Activity for Health: a consensus statement from the British Association of Sport and Exercise Sciences. Journal of sports sciences. 2010;28(6):573-91.
5. Campbell-Jack D, Hinchliffe S, Rutherford L, Brown L, Gray L, Hovald P, et al. The Scottish Health Survey 2015. Edinburgh: Scottish Government; 2015.
6. Townsend N, Wickramasinghe K, Williams J, Bhatnagar P, Rayner M, Nuffield Department of Population Health. British Heart Foundation - Physical Activity Statistics. 2015.
7. Farrally MR, Cochran AJ, Crews DJ, Hurdzan MJ, Price RJ, Snow JT, et al. Golf science research at the beginning of the twenty-first century. Journal of sports sciences. 2003;21(9):753-65.
8. The Royal and Ancient. Golf around the world. The Royal and Ancient, 2015.
9. England Golf. England Golf Membership Survey 2014. 2014.
10. Murray AD, Daines L, Archibald D, Hawkes RA, Schiphorst C, Kelly P, et al. The relationships between golf and health: a scoping review. British journal of sports medicine. 2017;51(1):12-9.
11. Ainsworth BE, Haskell WL, Herrmann SD, Meckes N, Bassett DR, Jr., Tudor-Locke C, et al. 2011 Compendium of Physical Activities: a second update of codes and MET values. Med Sci Sports Exerc. 2011;43(8):1575-81.
12. Haskell WL, Lee IM, Pate RR, Powell KE, Blair SN, Franklin BA, et al. Physical activity and public health: updated recommendation for adults from the American College of Sports Medicine and the American Heart Association. Medicine and science in sports and exercise. 2007;39(8):1423-34.
13. National Centre for Social Research. Health Survey for England. 2012.
14. American College of Sports Medicine., Pescatella LS, Arena R, Riebe D, Thompson PD. ACSM's guidelines for exercise testing and prescription. Ninth edition. ed. Baltimore: Lippincott Williams & Wilkins; 2013.
15. Murray AD, Daines L, Archibald D, Hawkes RA, Schiphorst C, Kelly P, et al. Infographic. Golf and health. British journal of sports medicine. 2017;51(1):20-1.
16. Peters M GC, McInerney P, Soares CB, Khalil H, Parker D. The Joanna Briggs Institute Reviewers' Manual 2015: Methodology for JBI Scoping Reviews. : The Joanna Briggs Institute; 2015.
17. Luscombe J, Murray A, Jenkins E, Archibald D. A rapid review to identify physical activity accrued whilst playing golf: PROSPERO 2017:CRD42017058237; [Available from: http://www.crd.york.ac.uk/PROSPERO/display_record.asp?ID=CRD42017058237.

For peer review only

For peer review only - http://bmjopen.bmj.com/site/about/guidelines.xhtml

18. Liberati A, Altman DG, Tetzlaff J, Mulrow C, Gotzsche PC, Ioannidis JP, et al. The PRISMA statement for reporting systematic reviews and meta-analyses of studies that evaluate healthcare interventions: explanation and elaboration. Bmj. 2009;339:b2700.
19. Watt A, Cameron A, Sturm L, Lathlean T, Babidge W, Blamey S, et al. Rapid versus full systematic reviews: validity in clinical practice? ANZ journal of surgery. 2008;78(11):1037-40.
20. Harker J, Kleijnen J. What is a rapid review? A methodological exploration of rapid reviews in Health Technology Assessments. Int J Evid Based Healthc. 2012;10.
21. Haby MM, Chapman E, Clark R, Barreto J, Reveiz L, Lavis JN. What are the best methodologies for rapid reviews of the research evidence for evidence-informed decision making in health policy and practice: a rapid review. Health Research Policy and Systems. 2016;14(1):83.
22. Effective Public Health Practice Project. Quality Assessment Tool for Quantitative Studies 2004 [Available from: http://www.ephpp.ca/PDF/Quality%20Assessment%20Tool_2010_2.pdf.
23. National Heart Lung and Blood Insitute. Quality Assessment Tool for Observational Cohort and Cross-Sectional Studies 2014 [Available from: https://www.nhlbi.nih.gov/health-pro/guidelines/in-develop/cardiovascular-risk-reduction/tools/cohort.
24. Burkett LN, von Heijne-Fisher U. Heart rate and calorie expenditure of golfers carrying their clubs and walking flat and hilly golf courses. International Sports Journal. 1998;2(2):78-85.
25. Crowell BG. Energy cost of participation in golf as determined by telemetry: Oklahoma State University; 1970.
26. Dear JB, Porter MM, Ready AE. Energy expenditure during golfing and lawn mowing in older adult men. J Aging Phys Act. 2010;18(2):185-200.
27. Dobrosielski DA, Brubaker PH, Berry MJ, Ayabe M, Miller HS. The metabolic demand of golf in patients with heart disease and in healthy adults. J Cardiopulm Rehabil. 2002;22(2):96-104.
28. Gabellieri JM. The physiological demands of walking during golf: University of Rhode Island; 2011.
29. Lampley JH, Lampley PM, Howley ET. Caloric cost of playing golf. Res Q. 1977;48(3):637-9.
30. Loy SF. The effect of the game of golf on cardiopulmonary fitness of middle-aged men: California State University, Northridge; 1979.
31. Murase Y, Kamei S, Hoshikawa T. Heart rate and metabolic responses to participation in golf. The Journal of sports medicine and physical fitness. 1989;29(3):269-72.
32. Zunzer SC, von Duvillard SP, Tschakert G, Mangus B, Hofmann P. Energy expenditure and sex differences of golf playing. Journal of sports sciences. 2013;31(10):1045-53.
33. Tangen JO, Sunde A, Sageie J, Hagen PC, Kristoffersen B, Istad R, et al. In accordance with governmental recommendations—a study of golf and health. Journal of sports sciences. 2013;1:15-25.
34. Moy K, Scragg R, McLean G, Carr H. Metabolic equivalent (MET) intensities of culturally-specific physical activities performed by New Zealanders. N Z Med J. 2006;119(1235):U2000.
35. Unverdorben M, Kolb M, Bauer I, Bauer U, Brune M, Benes K, et al. Cardiovascular load of competitive golf in cardiac patients and healthy controls. Medicine and science in sports and exercise. 2000;32(10):1674-8.
36. Broman G, Johnsson L, Kaijser L. Golf: a high intensity interval activity for elderly men. Aging clinical and experimental research. 2004;16(5):375-81.
37. Getchell LH. An Analysis of the Effects of a Season of Golf on Selected Cardiovascular, Metabolic, and Muscular Fitness Measures on Middle-aged Men; and the Caloric Cost of Golf: University of Oregon; 1965.
38. Kras J, Larsen B. A comparison of the health benefits of walking and riding during a round of golf. International Sports Journal. 2002;6(1):112-6.
39. Stauch M, Liu Y, Giesler M, Lehmann M. Physical activity level during a round of golf on a hilly course. The Journal of sports medicine and physical fitness. 1999;39(4):321-7.

For peer review only - http://bmjopen.bmj.com/site/about/guidelines.xhtml

40. Kobriger SL, Smith J, Hollman JH, Smith AM. The contribution of golf to daily physical activity recommendations: how many steps does it take to complete a round of golf? Mayo Clin Proc. 2006;81(8):1041-3.
41. Sell TC, Tsai YS, Smoliga JM, Myers JB, Lephart SM. Strength, flexibility, and balance characteristics of highly proficient golfers. J Strength Cond Res. 2007;21(4):1166-71.
42. Gao KL, Hui-Chan CW, Tsang WW. Golfers have better balance control and confidence than healthy controls. Eur J Appl Physiol. 2011;111(11):2805-12.
43. Tsang WW, Hui-Chan CW. Effects of exercise on joint sense and balance in elderly men: Tai Chi versus golf. Medicine and science in sports and exercise. 2004;36(4):658-67.
44. Tsang WW, Hui-Chan CW. Static and dynamic balance control in older golfers. J Aging Phys Act. 2010;18(1):1-13.
45. Schachten T, Jansen P. The effects of golf training in patients with stroke: a pilot study. Int Psychogeriatr. 2015;27(5):865-73.
46. Heyvaert M, Hannes K, Onghena P. Using Mixed Methods Research Synthesis for Literature Reviews: SAGE Publications, Inc; 2017.
47. Garber CE, Blissmer B, Deschenes MR, Franklin BA, Lamonte MJ, Lee IM, et al. American College of Sports Medicine position stand. Quantity and quality of exercise for developing and maintaining cardiorespiratory, musculoskeletal, and neuromotor fitness in apparently healthy adults: guidance for prescribing exercise. Medicine and science in sports and exercise. 2011;43(7):1334-59.
48. Sports Marketing Surveys Inc. 2014 Golf Participation. 2014.
49. Tudor-Locke C, Craig CL, Brown WJ, Clemes SA, De Cocker K, Giles-Corti B, et al. How many steps/day are enough? For adults. The international journal of behavioral nutrition and physical activity. 2011;8:79.

For peer review only

Appendix 1. Searching protocol, phase 1

Step 1

SPORT Discus

1st search: from 1900, all articles, all languages
Search for: Golf AND health
Hits: 1364

2nd search
Search for: Golf AND health AND review
Hits: 56, of which 11 relevant studies identified

Google Advanced

1st search: Golf AND health AND review
Hits:487,000
First 200 articles reviewed for relevance, 15 appeared relevant

ProQuest dissertations

1st search: Golf AND health
Hits: 4, of which 2 relevant covering narrow aspect of topic.

Reference section of useful studies reviewed.

Step 2

The main category – terms are:

1. Golf
   Plus/minus
2. Health OR illness OR injur* OR fitness OR mortality OR morbidity

SPORTDiscus

1st search: from 1900, all articles, all languages
Search for: Golf AND health OR illness OR injur* OR fitness OR mortality OR morbidity
Hits: 3781

2nd search: from 1900, all articles excluding magazine, all languages
Search for: Golf AND health OR illness OR injur* OR fitness OR mortality OR morbidity excluding magazines
Hits: 744

Web of Science

1st search: from 1900, all articles, all languages,
Search for: Golf AND health OR illness OR injur* OR fitness OR mortality OR morbidity

For peer review only

Hits: 559

PsycINFO

1st search from 1900, all articles, all languages
Search for: Golf
Topics Hits: 832

Medline

1st search: from 1900, all articles, all languages
Search for: Golf
Hits: 1721

Google scholar

1st search: from 1900, articles and patents, include citations
Search for: Golf AND health OR illness OR injury OR fitness OR mortality OR morbidity
Hits: 154000, >too many

2nd Search: from 1900, articles and patents, include citations
Search for: Golf AND health OR illness OR injury OR fitness OR mortality OR morbidity
Within title
Hits: 185

Initial database search

Hits: 4041 before duplicates
3167 once duplicates removed

Grey Literature

Google (advanced search)

British Journal of Sports Medicine Domain
1st search: from 1900, all articles, all languages
Search for: Golf AND health OR illness OR injury OR fitness OR mortality OR morbidity AND specify URL http://bjsm.bmj.com/
Hits: 548
https://www.google.co.uk/search?as_q=golf&as_epq=&as_oq=health+illness+injury++fitness++mortality+morbidity+&as_eq=&as_nlo=&as_nhi=&lr=&cr=&as_qdr=all&as_sitesearch=http://bjsm.bmj.com/&as_occt=any&safe=images&as_filetype=&as_right=&gws_rd=cr&ei=Eq1AVue-OsfTU6XPtOAC#q=golf+health+OR+illness+OR+injury+OR+fitness+OR+mortality+O R+morbidity+site:http://bjsm.bmj.com/&as_qdr=all&start=20

For peer review only

World Golf Foundation

1st search: from 1900, all articles, all languages

Search for: Golf AND health OR illness OR injury OR fitness OR mortality OR morbidity AND specify URL http://www.worldgolffoundation.org/

Hits:11

https://www.google.com/search?as_q=golf&as_epq=&as_oq=health+illness+injury+fitness+
mortality+morbidity+&as_eq=&as_nlo=&as_nhi=&lr=&cr=&as_qdr=all&as_sitesearch=http %3A%2F%2Fwww.worldgolffoundation.org%2F&as_occt=any&safe=images&as_filetype= &as_rights=

Royal and Ancient

1st search: from 1900, all articles, all languages

Search for: Golf AND health OR illness OR injury OR fitness OR mortality OR morbidity AND specify URL http://www.randa.org/

Hits: 133

https://www.google.com/search?as_q=golf&as_epq=&as_oq=health+illness+injury+fitness+
mortality+morbidity+&as_eq=&as_nlo=&as_nhi=&lr=&cr=&as_qdr=all&as_sitesearch=http %3A%2F%2Fwww.randa.org%2F&as_occt=any&safe=images&as_filetype=&as_rights=

Faculty of Sports and Exercise Medicine

1st search: from 1900, all articles, all languages

Search for: Golf AND health OR illness OR injury OR fitness OR mortality OR morbidity AND specify URL http://www.fsem.ac.uk/

Hits: 8

https://www.google.com/search?as_q=golf&as_epq=&as_oq=health+injury+illness+morbidit
y+mortality&as_eq=&as_nlo=&as_nhi=&lr=&cr=&as_qdr=all&as_sitesearch=http%3A%2F %2Fwww.fsem.ac.uk%2F&as_occt=any&safe=images&as_filetype=&as_rights=

American College for Sports Medicine

1st search: from 1900, all articles, all languages

Search for: Golf AND health OR illness OR injury OR fitness OR mortality OR morbidity AND specify URL http://www.acsm.org/

Hits: 26

https://www.google.com/search?as_q=golf&as_epq=&as_oq=health+injury+illness+morbidit
y+mortality&as_eq=&as_nlo=&as_nhi=&lr=&cr=&as_qdr=all&as_sitesearch=http%3A%2F %2Fwww.acsm.org%2F&as_occt=any&safe=images&as_filetype=&as_rights=

ProQuest dissertations

1st search: from 1900, all articles, all languages

Search for: Golf AND health OR illness OR injury OR fitness OR mortality OR morbidity

For peer review only

Hits: 42740 >too many

2nd search: Golf AND Health OR illness OR injur* OR fitness OR mortality OR morbidity in title or abstract
Hits: 175 Duplicates 115

World Health Organisation- International Clinical Trials Registry Platform

1st search
Search for: Golf
Hits: 2
http://apps.who.int/trialsearch/Trial2.aspx?TrialID=NCT02544399 (relevant)
http://apps.who.int/trialsearch/Trial2.aspx?TrialID=EUCTR2005-003458-81-IT (not relevant)

Initial Grey Literature Databases search

Hits: 903

For peer review only

Appendix 2. Searching protocol, phase 2

SPORTDiscus
From 1900, all articles, all languages
Search for: Golf AND health
Hits: 32

Web of Science
From 1900, all articles, all languages,
Search for: Golf AND health
Hits: 29

PsycInfo
From 1900, all articles, all languages
Search for: Golf
Hits: 10

Medline
From 1900, all articles, all languages
Search for: Golf
Hits: 91

Google Scholar
From 1900, articles and patents, include citations
Search for: Golf AND health OR illness OR injury OR fitness OR mortality OR morbidity
Within title
Hits: 8

For peer review only

Appendix 3. Sample Data Extraction Form.

Bibliographic Information

**Study ID:**	**Date of extraction:**	**Extracted by:**	**Checked by:**
**Year published:**	**Country:**		
**Title:**			
**Author(s):**			

Study Characteristics

**Aims/purpose:**
**Study design:**
**Analytical methods deployed:**
**Study procedures:**

Participants Characteristics

**Age:**	**Gender:**	**Sample size:**
**Any disease characteristics:**	**Handicap/Average score:**	

Intervention

**Intervention Type:**
**Comparator:**
**Setting:**

For peer review only

**Duration:**	
**Modifiers:**	
Sex	
Hills	
Golf Carts/Pulling/Carrying Clubs	
Age	
Other	

Outcomes

**METS**	
**Energy expenditure**	
**O_2 intake**	
**Heart rate**	
**Steps taken**	
**Distance covered**	
**Strength**	
**Flexibility**	
**Balance**	
**Sedentary Behaviour**	
**Outcome Measures:**	

Results

**Key findings:**
**Limitations:**

For peer review only

For peer review only

Appendix 4. Results of phase one of the systematic electronic search.

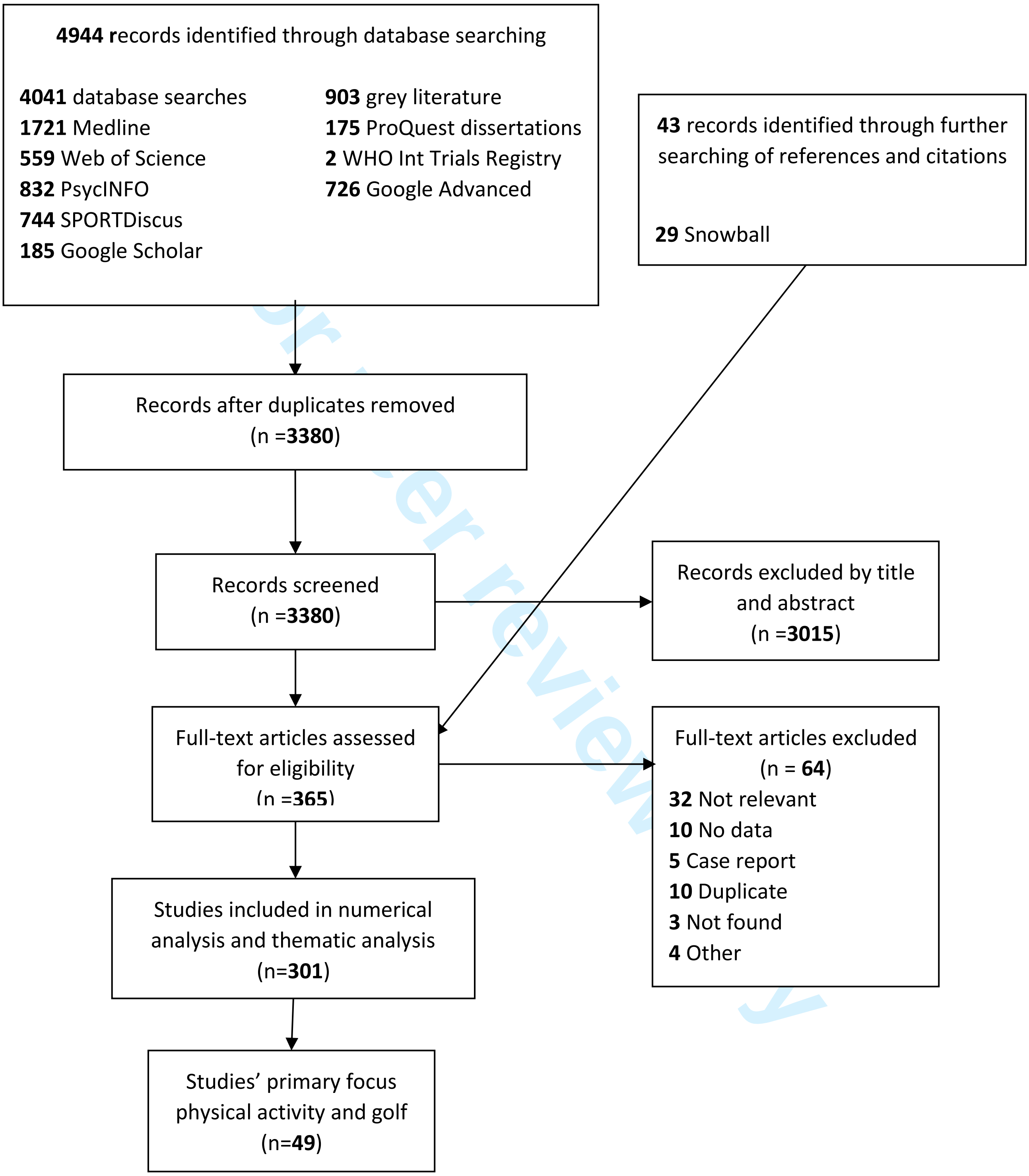

Adapted from Murray et al. [10]

Appendix 5. Citations of studies included in the rapid review

1. Burkett LN, von Heijne-Fisher U. Heart rate and calorie expenditure of golfers carrying their clubs and walking flat and hilly golf courses. International Sports Journal. 1998;2(2):78-85.

2. Broman G, Johnsson L, Kaijser L. Golf: a high intensity interval activity for elderly men. Aging clinical and experimental research. 2004;16(5):375-81.

3. Crowell BG. Energy cost of participation in golf as determined by telemetry: Oklahoma State University; 1970.

4. Dear JB, Porter MM, Ready AE. Energy expenditure during golfing and lawn mowing in older adult men. J Aging Phys Act. 2010;18(2):185-200.

5. Dobrosielski DA, Brubaker PH, Berry MJ, Ayabe M, Miller HS. The metabolic demand of golf in patients with heart disease and in healthy adults. J Cardiopulm Rehabil. 2002;22(2):96-104.

6. Gabellieri JM. The physiological demands of walking during golf: University of Rhode Island; 2011.

7. Gao KL, Hui-Chan CW, Tsang WW. Golfers have better balance control and confidence than healthy controls. Eur J Appl Physiol. 2011;111(11):2805-12.

8. Getchell LH. An Analysis of the Effects of a Season of Golf on Selected Cardiovascular, Metabolic, and Muscular Fitness Measures on Middle-aged Men; and the Caloric Cost of Golf: University of Oregon; 1965.

9. Kobriger SL, Smith J, Hollman JH, Smith AM. The contribution of golf to daily physical activity recommendations: how many steps does it take to complete a round of golf? Mayo Clin Proc. 2006;81(8):1041-3.

10. Kras J, Larsen B. A comparison of the health benefits of walking and riding during a round of golf. International Sports Journal. 2002;6(1):112-6.

11. Lampley JH, Lampley PM, Howley ET. Caloric cost of playing golf. Res Q. 1977;48(3):637-9.

12. Loy SF. The effect of the game of golf on cardiopulmonary fitness of middle-aged men: California State University, Northridge; 1979.

13. Moy K, Scragg R, McLean G, Carr H. Metabolic equivalent (MET) intensities of culturally-specific physical activities performed by New Zealanders. N Z Med J. 2006;119(1235):U2000.

14. Murase Y, Kamei S, Hoshikawa T. Heart rate and metabolic responses to participation in golf. The Journal of sports medicine and physical fitness. 1989;29(3):269-72.

15. Sell TC, Tsai YS, Smoliga JM, Myers JB, Lephart SM. Strength, flexibility, and balance characteristics of highly proficient golfers. J Strength Cond Res. 2007;21(4):1166-71.

For peer review only

16. Stauch M, Liu Y, Giesler M, Lehmann M. Physical activity level during a round of golf on a hilly course. The Journal of sports medicine and physical fitness. 1999;39(4):321-7.

17. Tsang WW, Hui-Chan CW. Effects of exercise on joint sense and balance in elderly men: Tai Chi versus golf. Medicine and science in sports and exercise. 2004;36(4):658-67.

18. Tsang WW, Hui-Chan CW. Static and dynamic balance control in older golfers. J Aging Phys Act. 2010;18(1):1-13.

19. Unverdorben M, Kolb M, Bauer I, Bauer U, Brune M, Benes K, et al. Cardiovascular load of competitive golf in cardiac patients and healthy controls. Medicine and science in sports and exercise. 2000;32(10):1674-8.

20. Zunzer SC, von Duvillard SP, Tschakert G, Mangus B, Hofmann P. Energy expenditure and sex differences of golf playing. Journal of sports sciences. 2013;31(10):1045-53.

21. Tangen JO, Sunde A, Sageie J, Hagen PC, Kristoffersen B, Istad R, et al. In accordance with governmental recommendations—a study of golf and health. Journal of sports sciences. 2013;1:15-25.

22. Schachten T, Jansen P. The effects of golf training in patients with stroke: a pilot study. Int Psychogeriatr. 2015;27(5):865-73.

For peer review only

Appendix 6. Characteristics of included studies

Study ID	Author, publication year	Country	Sample size	Participants' age (mean years)	Participants' gender (% Male)	Participants' handicap or average score (mean strokes)	Participant's disease characteristics	Duration	Modifiers	Relevant outcome measures
1	Burkett, 1998	USA	10	24.0	100	<80[†] (50%)<br>80-95[†] (50%)	Healthy	3 x 18 holes	Course profile; skill level	Energy expenditure; heart rate
3	Broman, 2004	Sweden	19	27.0 (31%)<br>50.0 (37%)<br>75.0 (31%)	100	NR	1 x Polymyalgia rheumatica; 7 x "back problems"; 2 x knee pain	18 holes	Age; areas of golf course	Heart rate
4	Crowell, 1970	USA	7	42.7	100	11.6	NR	3 x 9 holes	Club transportation; shot type	Energy expenditure; oxygen intake; heart rate; distance covered
7	Dear, 2010	USA	18	71.2	100	NR	Healthy	9 holes	-	METs; energy expenditure; oxygen intake; heart rate; distance covered
8	Dobrosielski, 2002	USA	20	63.6 (50%)<br>65.8 (50%)	100	NR	2 x myocardial infarction<br>4 x coronary artery bypass graft<br>2 x percutaneous coronary intervention<br>1 x cardiomyopathy	9 holes	Heart disease	METs; energy expenditure

For peer review only

11	Gabellieri, 2011	USA	13	28.5	100	97.1†	NR	18 holes	Weight	METs; energy expenditure; heart rate; steps taken; distance covered
12	Gao, 2011	China	23	66.2 (47.8%) 71.3 (52.2%)	100	NR	Healthy	NA	-	Balance
14	Getchell, 1965	USA	29	39.4 (69.0%) 38.0 (31.0%)	100	78†	Sedentary lifestyle	4-8 months between initial test and retest	-	Oxygen intake; heart rate; strength; flexibility
24	Kobriger, 2006	USA	42	55	28.6	NR	NR	3 x 18 holes	Sex	Steps taken
25	Kras, 2002	USA	12	42-57	100	7-21	NR	3 x 9 holes	Course profile, club transportation	Heart rate
26	Lampley, 1977	USA	22	34 (50%) 32 (50%)	50	19	NR	9 holes	Sex	Energy expenditure
27	Loy, 1979	USA	6	52.5	100	15.7	Healthy	18 holes	-	Energy expenditure; oxygen intake; heart rate
30	Moy, 2006	New Zealand	2	NR	NR	NR	NR	NR	-	METs
31	Murase, 1989	Japan	5	30-50	100	90-100†	Healthy	18 holes	-	Energy expenditure; oxygen intake; heart rate
34	Sell, 2008	USA	257	45.5	100	<0 (17.5%) 0-9 (46.7%) 10-20 (35.8%)	NR	NA	Skill level	Strength; balance; flexibility
35	Stauch, 2003	USA	30	53	70	29	9 x cardiovascular disease	18 holes	Course profile; sex	Heart rate

For peer review only

37	Tsang, 2004	China	35	69.6 (34.3%)<br>66.2 (31.4%)<br>71.3 (34.3%)<br>20.3 (34.3%)	100	NR	NR	NA	-	Balance
38	Tsang, 2010	China	23	66.2 (47.8%)<br>71.3 (52.2%)	100	NR	NR	NA	-	Balance
39	Unverdorben, 2000	Germany	28	65.3 (71.4%)<br>62.0 (28.6%)	100	NR	11 x coronary artery disease; 10 x post myocardial infarction; 2 x peripheral arterial occlusive disease; 14 x arrhythmias; 1 x post-myocarditis; 2 x valve disease; 1 x diabetes mellitus; 12 x hypertension	18 holes	Heart disease	METs; oxygen intake; heart rate
41	Zunzer, 2013	Austria	66	53.3	63.6	26.4 (51.5%)<br>20.4 (48.5%)	NR	9 holes or 18 holes	Sex; course profile; club transportation; age	METs; energy expenditure; heart rate; distance covered
49	Tangen, 2013	Norway	29	44.2	48.3	27.3	NR	18 holes	Sex; course profile; age; skill level	METs; energy expenditure; heart rate; steps taken; distance covered
50	Schachen, 2015	Germany	14	55.1 (50%)<br>53.1 (50%)	NR	NR	Stroke	10 weeks (2 putting session/w	-	Balance

For peer review only

								eek)		

NR: Not reported
NA: not applicable
**calculated*
†*average score*

For peer review only

Appendix 7. Quality assessment of included studies.

Author, year	Clear objective	Clearly defined study population	Participation rate of eligible persons >50%	Recruited from same or similar populations + inclusion and exclusion criteria prespecified	Sample size justification, power description, or variance and effect estimates	Exposures measured prior to outcome being measured	Sufficient timeframe	Different levels of exposure examined	Exposure measures clearly defined, valid, reliable and implemented consistently	Exposure assessed more than once over time	Outcome measures clearly defined, valid, reliable and implemented consistently	Outcome assessors blinded to exposure status	Loss to follow-up <20%	Key confounding variables measured and adjusted for	Overall Rating
Burkett, 1998	Y	N	NA	CD	N	NA	Y	Y	N	NA	Y	CD	NA	N	**Fair**
Broman, 2004	Y	Y	NA	Y	N	NA	Y	N	N	NA	Y	NA	NA	N	**Fair**
Crowell, 1970	Y	Y	NA	N	N	NA	Y	Y	Y	NA	Y	CD	NA	Y	**Fair**
Dear, 2010	Y	Y	NA	Y	N	NA	Y	N	N	NA	Y	CD	NA	N	**Fair**
Dobrosielski, 2002	Y	Y	NA	N	N	NA	Y	N	N	NA	Y	CD	NA	N	**Fair**
Gabellieri, 2011	Y	N	NA	CD	Y	NA	Y	N	Y	N	Y	NA	NA	N	**Fair**
Gao, 2011	Y	Y	NA	Y	Y	NA	Y	N	Y	NA	Y	CD	NA	Y	**Good**
Getchell, 1965	Y	Y	NA	Y	Y	N	Y	CD	N	N	Y	CD	Y	N	**Poor**
Kobriger, 2006	Y	N	NA	Y	Y	NA	Y	Y	N	N	Y	NA	NA	N	**Fair**
Kras, 2002	Y	N	NA	CD	Y	NA	Y	Y	Y	N	Y	CD	NA	N	**Fair**
Lampley, 1977	Y	N	NA	CD	N	NA	Y	N	N	N	Y	NA	NA	Y	**Fair**
Lay, 1979	Y	Y	NA	N	N	NA	Y	N	N	N	Y	NA	NA	N	**Fair**
Moy, 2006	Y	N	NA	N	N	NA	CD	CD	N	N	Y	NA	NA	N	**Poor**
Murase, 1989	Y	N	NA	CD	N	NA	Y	N	Y	N	Y	NA	NA	N	**Poor**

For peer review only

Sell, 2008	Y	N	NA	CD	N	Y	CD	Y	Y	NA	Y	CD	NA	N	**Good**
Stauch, 2003	Y	Y	NA	N	N	NA	Y	Y	Y	NA	Y	NA	NA	N	**Fair**
Tsang, 2004	Y	N	NA	N	N	Y	Y	N	Y	N	Y	CD	NA	Y	**Fair**
Tsang, 2010	Y	Y	NA	Y	N	Y	Y	N	Y	N	Y	CD	NA	Y	**Good**
Unverdorben, 2000	Y	Y	NA	N	N	NA	Y	N	Y	N	Y	CD	NA	Y	**Good**
Zunzer, 2013	Y	Y	NA	Y	N	NA	Y	Y	Y	N	Y	CD	NA	Y	**Good**
Tangen, 2013	Y	N	NA	CD	N	NA	Y	Y	N	N	Y	CD	NA	Y	**Fair**
Schachen, 2015	Y	N	NA	N	Y	NA	Y	N	Y	NA	Y	CD	NA	N	**Fair**

Y: yes
N: no
NA: not applicable
CD: can't determine
NR: not reported

PRISMA 2009 Checklist

Section/topic	#	Checklist item	Reported on page #
**TITLE**			
Title	1	Identify the report as a systematic review, meta-analysis, or both.	0
**ABSTRACT**			
Structured summary	2	Provide a structured summary including, as applicable: background; objectives; data sources; study eligibility criteria, participants, and interventions; study appraisal and synthesis methods; results; limitations; conclusions and implications of key findings; systematic review registration number.	1
**INTRODUCTION**			
Rationale	3	Describe the rationale for the review in the context of what is already known.	2
Objectives	4	Provide an explicit statement of questions being addressed with reference to participants, interventions, comparisons, outcomes, and study design (PICOS).	2
**METHODS**			
Protocol and registration	5	Indicate if a review protocol exists, if and where it can be accessed (e.g., Web address), and, if available, provide registration information including registration number.	2
Eligibility criteria	6	Specify study characteristics (e.g., PICOS, length of follow-up) and report characteristics (e.g., years considered, language, publication status) used as criteria for eligibility, giving rationale.	3-4
Information sources	7	Describe all information sources (e.g., databases with dates of coverage, contact with study authors to identify additional studies) in the search and date last searched.	3
Search	8	Present full electronic search strategy for at least one database, including any limits used, such that it could be repeated.	Appendices 1 + 2
Study selection	9	State the process for selecting studies (i.e., screening, eligibility, included in systematic review, and, if applicable, included in the meta-analysis).	3-4
Data collection process	10	Describe method of data extraction from reports (e.g., piloted forms, independently, in duplicate) and any processes for obtaining and confirming data from investigators.	4
Data items	11	List and define all variables for which data were sought (e.g., PICOS, funding sources) and any assumptions and simplifications made.	4
Risk of bias in individual studies	12	Describe methods used for assessing risk of bias of individual studies (including specification of whether this was done at the study or outcome level), and how this information is to be used in any data synthesis.	4
Summary measures	13	State the principal summary measures (e.g., risk ratio, difference in means).	4
Synthesis of results	14	Describe the methods of handling data and combining results of studies, if done, including measures of consistency (e.g., I^2) for each meta-analysis.	4

For peer review only

For peer review only - http://bmjopen.bmj.com/site/about/guidelines.xhtml

PRISMA 2009 Checklist

Section/topic	#	Checklist item	Reported on page #
Risk of bias across studies	15	Specify any assessment of risk of bias that may affect the cumulative evidence (e.g., publication bias, selective reporting within studies).	NA
Additional analyses	16	Describe methods of additional analyses (e.g., sensitivity or subgroup analyses, meta-regression), if done, indicating which were pre-specified.	NA
**RESULTS**			
Study selection	17	Give numbers of studies screened, assessed for eligibility, and included in the review, with reasons for exclusions at each stage, ideally with a flow diagram.	4-5
Study characteristics	18	For each study, present characteristics for which data were extracted (e.g., study size, PICOS, follow-up period) and provide the citations.	5-6
Risk of bias within studies	19	Present data on risk of bias of each study and, if available, any outcome level assessment (see item 12).	6
Results of individual studies	20	For all outcomes considered (benefits or harms), present, for each study: (a) simple summary data for each intervention group (b) effect estimates and confidence intervals, ideally with a forest plot.	6-13
Synthesis of results	21	Present results of each meta-analysis done, including confidence intervals and measures of consistency.	NA
Risk of bias across studies	22	Present results of any assessment of risk of bias across studies (see Item 15).	6
Additional analysis	23	Give results of additional analyses, if done (e.g., sensitivity or subgroup analyses, meta-regression [see Item 16]).	NA
**DISCUSSION**			
Summary of evidence	24	Summarize the main findings including the strength of evidence for each main outcome; consider their relevance to key groups (e.g., healthcare providers, users, and policy makers).	14-15
Limitations	25	Discuss limitations at study and outcome level (e.g., risk of bias), and at review-level (e.g., incomplete retrieval of identified research, reporting bias).	15
Conclusions	26	Provide a general interpretation of the results in the context of other evidence, and implications for future research.	15
**FUNDING**			
Funding	27	Describe sources of funding for the systematic review and other support (e.g., supply of data); role of funders for the systematic review.	15

From: Moher D, Liberati A, Tetzlaff J, Altman DG, The PRISMA Group (2009). Preferred Reporting Items for Systematic Reviews and Meta-Analyses: The PRISMA Statement. PLoS Med 6(7): e1000097. doi:10.1371/journal.pmed1000097

For more information, visit: **www.prisma-statement.org**.

For peer review only - http://bmjopen.bmj.com/site/about/guidelines.xhtml

BMJ Open

A rapid review to identify physical activity accrued whilst playing golf

Journal:	*BMJ Open*
Manuscript ID	bmjopen-2017-018993.R1
Article Type:	Research
Date Submitted by the Author:	05-Oct-2017
Complete List of Authors:	Luscombe, Jack; University of Edinburgh, Physical Activity for Health Research Centre; University of Edinburgh, Medical School<br>Murray, Andrew; University of Edinburgh, Physical Activity for Health Research Centre; University of Edinburgh, Sport and Exercise<br>Jenkins, Evan; University of Edinburgh, Physical Activity for Health Research Centre; University of Edinburgh, Medical School<br>Archibald, Daryll; University of Edinburgh, Scottish Collaboration for Public Health Research and Policy
**Primary Subject Heading**:	Public health
Secondary Subject Heading:	Sports and exercise medicine
Keywords:	golf, physical activity, health

SCHOLARONE™
Manuscripts

Title Page

A rapid review to identify physical activity accrued whilst playing golf

Luscombe J[1, 2], Murray AD[2, 3], Jenkins E[1, 2], Archibald D[4]

Corresponding author:

Jack Luscombe, Physical Activity for Health Research Centre, Room 2.33 St Leonard's Land, University of Edinburgh, Edinburgh, UK. EH8 8AQ

Email: s1204551@sms.ed.ac.uk

Telephone: (+44)7772478162

Author affiliations:

[1]Medical School, University of Edinburgh, Edinburgh, United Kingdom.

[2]Physical Activity for Health Research Centre, University of Edinburgh, Edinburgh, United Kingdom.

[3]Sport and Exercise, University of Edinburgh, Edinburgh, United Kingdom.

[4]Scottish Collaboration for Public Health Research and Policy, University of Edinburgh, Edinburgh, United Kingdom.

For peer review only

Keywords: golf, physical activity, health

Word Count: 3535

ABSTRACT

Objective: To identify physical activity accrued whilst playing golf, and modifiers of physical activity accrued.

Design: A rapid review of primary research studies. Quality was assessed using the National Heart, Lung, and Blood Institute quality assessment tool for cohort and cross-sectional studies.

Methods and outcomes: The following databases were searched from 1900 to March 2017: SPORTDiscus, Web of Science, PsycINFO, MEDLINE, Google Scholar, Google Advanced Search, ProQuest, World Health Organisation International Clinical Trials Registry Platform. All primary research investigating golf or golfers with any of the following outcomes was included: metabolic equivalent of task, oxygen uptake, energy expenditure, heart rate, step count, distance covered, strength, flexibility, balance, sedentary behaviour.

Results: Phase one searching identified 4944 citations and phase two searching identified 170 citations. In total 19 articles met inclusion criteria. Golf is primarily a moderate intensity physical activity, but may be low intensity depending upon the playing population and various modifiers. Less physical activity is accrued by those who ride a golf cart compared to those walking the course.

Conclusions: Golf can be encouraged in order to attain physical activity (PA) recommendations. Further research is required into the relationship between golf and strength and flexibility physical activity recommendations, and how modifiers affect physical activity accrued.

Systematic review registration: PROSPERO 2017:CRD42017058237

ARTICLE SUMMARY

Strengths and limitations of this study

- This study is, to our knowledge, the first systematically conducted review to focus on golf and physical activity
- A comprehensive overview of golf and physical activity
- Rapid review – streamlined methods are not subject to the same rigor as a systematic review
- There was limited evidence with regards to strength and flexibility outcome measures

For peer review only - http://bmjopen.bmj.com/site/about/guidelines.xhtml

INTRODUCTION

Moderate intensity physical activity is known to provide longevity, physical and mental health benefits[1-4]. Physical activity guidelines[1] generally recommend, for adults, at least 150 minutes of moderate intensity activity, or 75 minutes of vigorous physical activity per week, or a combination of the two. In addition, physical activity to improve muscle strength on at least two days a week and efforts to minimise the amount of time spent sedentary are recommended. An estimated 41-51% of women and 32-41% of men do not meet these guidelines[5, 6] in the United Kingdom (UK). Furthermore, the proportion of adults meeting guidelines decreases with age - only 7-36% of adults aged 75 and over meet the recommendations[5, 6].

Golf is a popular sport played by over 50 million people[7] of all ages and abilities in over 200 countries[8]. In contrast to most sports, participation is higher in middle-aged and older adults[9-11]. Reviews and guideline documents have suggested golf can provide moderate intensity[1, 12-14] and muscle-strengthening physical activity[13]. These studies have not formally assessed the quality of the evidence.

The frequently-cited Compendium of Physical Activities[11] is a classification of intensity costs of various physical activities. It lists golf as, on average, providing 4.8 metabolic equivalents of task of physical activity, a moderate intensity.

A recently published systematically conducted scoping review[10, 15] provided an overview of golf and health and further highlighted that golf can provide moderate intensity physical activity. As per standard guidelines for undertaking scoping reviews[16], the relative strengths and limitations of included studies were not assessed. There have been no other reviews found that utilise systematic methods exploring physical activity and golf. We therefore aimed to provide a rapid review to identify physical activity accrued whilst playing golf.

Murray et al's[10] scoping review noted several factors that influence the intensity of physical activity whilst playing golf: use of a golf-cart, course profile, age, weight, sex, and baseline fitness of participants[10]. Our secondary aim was therefore to report modifiers to the amount of physical activity accrued whilst playing golf.

METHODS

Our systematic review adhered to our published protocol[17] and followed Preferred Reporting Items for Systematic Reviews and Meta-Analyses (PRISMA) guidelines[18].

Rapid reviews are a streamlined, time efficient and pragmatic approach to synthesise evidence. They have been shown to produce similar conclusions to systematic reviews[19]. Variable methodologies have been described[20], and therefore guidance was sought as to the best approach[21]. A rapid review was conducted due to a short time-frame in which to complete the research. To streamline the process, the search strategy from a recent scoping review[10] was used and adapted, there were less exhaustive searches of grey literature and only one reviewer assessed all papers for selection, data extraction and quality

For peer review only - http://bmjopen.bmj.com/site/about/guidelines.xhtml

assessment compared to a full systematic review. Similar concessions have been described in the literature[22].

Search strategy

We adopted a two-phase search strategy. The first phase employed the search strategy used in the recently published scoping review published by team members[10] – a precursor to this rapid review. The scoping review search was undertaken in November 2015 across the following databases: SPORTDiscus, Web of Science, PsycINFO, MEDLINE, Google Scholar, Google Advanced Search, ProQuest dissertations, World Health Organisation International Clinical Trials Registry Platform. The search identified 301 studies relating to the scoping review's aims – the relationship and effects of golf on physical and mental health. Forty-nine of those studies were found to be specifically related to golf and physical activity, which will be used in the current review.

The second phase of the search strategy involved adapting and updating the scoping review search. The search was re-run restricting its scope to search for papers related to golf and physical activity only, that were published from November 2015 to March 2017. A focussed grey literature search was performed using the modified terms 'golf AND health'. The full search strategies can be found in Appendices 1 and 2.

Study selection

One reviewer (JL) selected studies for review first by title and abstract, then by full text review, against inclusion/exclusion criteria with the exception of title and abstract screening of phase two results, conducted by DA. A second reviewer (EJ) independently reviewed a random sample of 10% of the papers by full text review for inclusion or exclusion. Concordance was checked and any discrepancies were discussed and resolved by a team member, either AM or DA.

Inclusion and exclusion criteria were developed through researcher discussion:

Inclusion criteria

- Research articles not limited by geographical location, language or setting
- Research articles published since 1900 up until March 2017
- Research articles discussing any of the following outcomes in relation to golf: metabolic equivalent of task (MET), oxygen uptake, energy expenditure, heart rate, step count, distance covered, strength, flexibility, balance, sedentary behaviour
- Any form of playing golf (including but not limited to 18 holes, nine holes, driving range) or research involving golfers
- All ages groups and both sexes of participants
- Sources of information including randomised control trials, cohort, case-control and cross-sectional studies, that have been synthesised quantitatively

Exclusion criteria

For peer review only - http://bmjopen.bmj.com/site/about/guidelines.xhtml

- Studies focussing exclusively on caddies and/or spectators
- Qualitative studies, reviews, opinion pieces, magazine and newspaper articles, case reports, conference proceedings.

Data extraction

Data were extracted by one reviewer (JL) using a data extraction form. The data extraction form was piloted using 10% of papers and modifications were made. A further random sample of 10% was independently extracted by a second reviewer (EJ) and results compared. Concordance was checked and any discrepancies were discussed and resolved by a team member, either AM or DA. A sample data extraction form can be found in Appendix 3.

Quality assessment

Our protocol[17] details use of the Effective Public Health Practice Project's quality assessment tool for quantitative studies[23] to assess study quality. After trialling, it became apparent the tool was more suited to interventional studies with groups. As the large majority of included studies are observation cross-sectional design, the tool was not suitable and therefore the National Heart, Lung, and Blood Institute quality assessment tool for observational cohort and cross-sectional studies[24] was used. Eligible studies were assessed by one reviewer (JL). A second reviewer (EJ) independently assessed a random sample of 10% of the papers using the same tool. Concordance was checked and any discrepancies were discussed and resolved by a third researcher, either AM or DA. Studies rated 'Fair' or 'Good' were included in the review.

Data synthesis and analysis

Due to the wide heterogeneity of included studies in terms of study design, population, setting, outcomes and study quality, data were synthesised narratively with summary tables and figures using the following outcomes: metabolic equivalent of task (MET), energy expenditure, oxygen uptake, heart rate, steps taken, distance covered, strength, flexibility, balance and sedentary behaviour. Modifiers to physical activity accrued were noted during data extraction and were also narratively synthesised. There were no principal summary measures due to the studies' heterogeneity; data were presented using the raw outcome measures.

RESULTS

Study Selection

In total, 3550 independent records were identified through our systematic two-phase electronic search. Three thousand three hundred and eighty independent records were identified in phase one[10]. Three thousand and fifteen records were excluded on screening of title and abstract, and 64 articles were excluded by full text review. Of the remaining 301 articles, 49 were specifically related to golf and physical activity. A flowchart detailing the results of phase one of the search can be found in Appendix 4.

For peer review only - http://bmjopen.bmj.com/site/about/guidelines.xhtml

Phase two of the search identified 170 further independent records (Figure 1). One hundred and sixty-eight records were excluded by title and abstract. The 49 articles from phase one were included here and assessed for eligibility by full text review. Thirty-two articles were excluded by full text review. Nineteen articles remained that met the inclusion criteria and were included in the review. Citations of included studies can be found in Appendix 5.

Study Characteristics

Of the 19 included studies, 11 were conducted in United States, 3 in China and 5 in other countries (Germany, Sweden, Austria and Norway). All included studies were cross sectional design. Sixteen of the identified studies were primary published research papers, 3 were published dissertations. The studies' publication dates ranged from 1970 – 2015. Four of the studies were published pre-2000, 15 studies were published post-2000.

Ten different outcome measures were used in the review. The most frequently reported were: heart rate (10 studies), energy expenditure (9 studies) and METs (6 studies). No studies reported on sedentary behaviour. Further characteristics of included studies are presented in Appendix 6.

Quality of Included Studies

Information on quality assessment of included studies can be found in Appendix 7. All studies provided a clear objective or research question. Most studies (74%) did not provide a sample size justification, power description or variance/effect estimates. It was unclear in the majority of studies whether outcome assessors were blinded to exposure status of participants (68%). Five studies were rated 'Good' and 14 were rated 'Fair'.

Outcomes

Energy Expenditure

Nine studies identified energy expenditure (EE) as an outcome[25-33]. Eight studies were rated 'Fair'[25-31, 33] and 1 rated 'Good'[32]. Results are detailed in Table 1. Two studies found significantly higher energy expenditure on hillier courses compared to flatter courses. Zunzer et al.[32], however, found no significant difference in energy expenditure between hilly and flat courses.

Lampley et al.[30] noted a significantly higher rate of energy expenditure in women. In contrast, two studies[32, 33] found males expended significantly more energy than females. However, Zunzer et al.[32] noted that this is not significant if body mass is accounted for and Tangen et al.[33] suggested this may be due to differences in course distance.

Two studies[25, 33] found no significant difference in energy expenditure in relation to skill level, despite less skilled players taking a larger number of shots in total, and on average being less able to advance the ball accurately

Crowell[26] noted the lowest energy expenditure when riding a golf cart, then pulling clubs and highest when carrying clubs. Zunzer et al.[32] found those who rode a

For peer review only - http://bmjopen.bmj.com/site/about/guidelines.xhtml

golf cart had significantly lower energy expenditure than those who pulled or carried clubs. Tangen et al.[33] found no significant difference in relation to club transportation; however it is noted that this may be due to small sample size in each group.

For peer review only

For peer review only - http://bmjopen.bmj.com/site/about/guidelines.xhtml

**Study**	**Quality Assessment**	**No. of Holes**	**Club Transportation**	**Course Profile**	**EE** *(kcal·min^{-1})*	**Net EE** *(kcal)*	**Gross EE** *(kcal)*	**EE** *(kcal·kg·hr^{-1})*
Burkett et al.[25]	Fair	18	Carrying clubs	Flat<br>Medium<br>Hilly	7.25 ± 1.75<br>8.15 ± 1.79<br>8.25 ± 1.83	-	-	-
Crowell[26]	Fair	9	Riding a golf cart<br>Pulling clubs<br>Carrying clubs	*Not reported*	5.2<br>6.8<br>7.5	-	-	-
Dear et al.[27]	Fair	9	Pulling clubs	*Not reported*	-	310.3 ± 83.9	511.6 ± 115.5	-
Dobrosielski et al.[28]	Fair	9	Pulling clubs	Mixed	-	458	-	--
Gabellieri[29]	Fair	18	Carrying clubs	“Undulating”	-	-	1202.8 ± 465.2	
Lampley et al.[30]	Fair	9	Pulling clubs	*Not reported*	-	-	-	4.2 ± 0.6 *(male)*<br>4.8 ± 0.4 *(female)*
Loy[31]	Fair	18	Carrying clubs	Hilly	6.2 ± 0.6	-	-	4.8*
Zunzer et al.[32]	Good	9<br><br><br>18	Mixed	Mixed	-	-	520 ± 133 *(male)*<br>273 ± 66 *(female)*<br>926 ± 292 *(male)*<br>556 ± 180 *(female)*	-
Tangen et al.[33]	Fair	18	Mixed	Hilly	-	-	2467 *(male)*<br>1587 *(female)*	-

Table 1. Energy expenditure for a round of golf.

*calculated for a 68kg man

Please refer to Appendix 6 for characteristics of the above studies.

For peer review only - http://bmjopen.bmj.com/site/about/guidelines.xhtml

Metabolic Equivalent of Task

Six studies identified metabolic equivalent of task (MET) as an outcome[27-29, 32-34]. Four of the studies were rated 'Fair'[27-29, 33] and 2 rated 'Good'[32, 34]. Results are detailed in Table 2. Dobrosielski et al.[28] found a significant difference between cardiac patients and healthy adults in average MET (57 ± 2.7; 46 ± 2.6% peak MET) and peak MET (89 ± 3.3; 77 ± 3.6% peak MET). However, Unverdorben et al.[34] found the same MET value (3.1) for cardiac patients and healthy adults. Zunzer et al.[32] noted no significant difference in METs between sexes; whereas Tangen et al.[33] found an almost significant difference between men and women (p=0.069). Zunzer et al.[32] found no significant difference in METs between hilly and flat golf courses.

**Study**	**Quality Assessment**	**No. of Holes**	**Club Transportation**	**METs** *(mean ± SD)*
Dear et al.[27]	Fair	9	Pulling clubs	2.8 ± 0.5
Dobrosielski et al.[28]	Fair	9	Pulling clubs	4.1 ± 0.1 *(cardiac disease)*
Gabellieri[29]	Fair	18	Carrying clubs	8.6 ± 3.1
Unverdorben et al.[34]	Good	18<br><br>18	Pulling clubs<br><br>Pulling clubs	3.1 *(cardiac disease)*<br>3.1 *(controls)*
Zunzer et al.[32]	Good	9<br><br>18	Mixed<br><br>Mixed	2.9 ± 0.8 *(male)*<br>2.2 ± 0.6 *(female)*<br>2.8 ± 0.7 *(male)*<br>2.1 ± 0.7 *(female)*
Tangen et al.[33]	Fair	18	Mixed	5.8 *(male)*<br>4.9 *(female)*

Table 2. Metabolic equivalent of task of a round of golf.

Please refer to Appendix 6 for characteristics of the above studies.

Heart Rate

Ten studies reported heart rate (HR) as an outcome[25, 26, 29, 31-37]. Eight were rated 'Fair'[25, 26, 29, 31, 33, 35-37] and 2 rated 'Good'[32, 34]. Mean HR and mean percentage of maximum HR (%HR_{max}) are presented in Table 3. In relation to maximum HR, Stauch et al.[35] found most time during a round of golf is spent at 50-74%HR_{max}. Tangen et al.[33] described 75% of a golf round is played at <70%HR_{max} and 25% is >70%. Broman et al.[35] found 70% of total time for elderly men is at >70%HR_{max}; whereas, for middle-aged and younger men, most time is spent at <70%HR_{max}. Loy[31] estimated 75.25 minutes are >60% heart rate reserve.

One paper[25] noted a significant difference in mean heart rate and a second paper[36], time spent >40%HR_{max} between hillier and flatter courses. Two papers found no significant difference in mean HR in relation to course profile[32, 33]. However, Tangen et al.[33] found a significantly higher maximum HR on the hillier course.

For peer review only - http://bmjopen.bmj.com/site/about/guidelines.xhtml

Two papers found highest heart rates when carrying clubs, then pulling clubs and lowest when riding a golf cart[26, 36]. One of these studies[36] found a significant difference in percentage of time spent >40%HR_{max} between carrying and pulling clubs, and riding a golf cart. Similarly, Zunzer et al.[32] found participants who rode a golf cart had significantly lower mean HR than those who carried or pulled their clubs. Stauch et al.[37] observed no significant difference in mean or maximum HR in relation to club transportation. However, it is noted there are significant differences in ages, a possible modifier to physical activity attained, between groups – this was also observed in another study[32].

Crowell[26] noted little difference in mean HR in relation to skill level and Burkett et al.[25] found no significant difference. In relation to sex, two papers[32, 33] observed no significant difference in mean HR and one paper[32], minimum, maximum HR or mean percentage HR_{max}. Broman et al.[35] found older golfers spent significantly more time at higher %HR_{max} than middle-aged or younger golfers. Tangen et al.[33] found older golfers (>50yrs) spent less time at high intensity level (>120bpm) than younger golfers (<50yrs) – but suggested this may be due to differences in maximum HR. Unverdorben et al.[34] observed no significant difference in mean HR between cardiac patients and healthy controls; but noted the maximum HR of controls was higher and therefore cardiac patients may work harder.

For peer review only - http://bmjopen.bmj.com/site/about/guidelines.xhtml

Study	Quality Assessment	No. of Holes	Club transportation	Course Profile	Mean HR (bpm)	Mean %HRmax
Burkett et al.[25]	Fair	18	Carrying clubs	Flat<br><br>Medium<br><br>Hilly	108.20 ± 13.16 *(GS)*<br>110.80 ± 7.26 *(AS)*<br>121.80 ± 18.54 *(GS)*<br>117.80 ± 13.54 *(AS)*<br>123.80 ± 21.81 *(GS)*<br>116.20 ± 14.97 *(AS)*	-
Crowell[26]	Fair	9	Riding a golf cart<br>Pulling clubs<br>Carrying clubs	*Not reported*	89.1 ± 10.6<br>103 ± 9.2<br>113.1 ± 8.8	-
Gabellieri[29]	Fair	18	Carrying clubs	"Undulating"	103.5 ± 13.2	55.2 ± 7.4
Loy[31]	Fair	18	Carrying clubs	Hilly	124.7 ± 8.6	
Stauch et al.[37]	Fair	18	Riding a golf cart<br>Pulling clubs<br>Carrying clubs	Hilly	111.0 ± 14.0<br>107.2 ± 11.0<br>118.4 ± 17.0	-
Unverdorben et al.[34]	Good	18	Pulling clubs	Hilly	105.4 ± 10.6 *(cardiac patients)*<br>100.5 ± 7.3 *(controls)*	-
Zunzer et al.[32]	Good	9<br><br>18	Mixed	Mixed	101 ± 12 *(male)*<br>99 ± 13 *(female)*<br>105 ± 14 *(male)*<br>103 ± 12 *(female)*	59.2 ± 3.1 *(male)*<br>59.2 ± 8.9 *(female)*<br>60.9 ± 8.6 *(male)*<br>61.6 ± 7.7 *(female)*
Tangen et al.[33]	Fair	18	Mixed	Hilly	104.1 ± 14.5 *(male)*<br>110.8 ± 16.9 *(female)*	-

Table 3. Mean heart rate and percentage of maximum heart rate during a round of golf.

GS: good skill (score<80)

AS: average skill (score 80-95)

Please refer to Appendix 6 for characteristics of the above studies.

For peer review only - http://bmjopen.bmj.com/site/about/guidelines.xhtml

For peer review only

Oxygen Uptake

Four studies listed oxygen uptake as an outcome[26, 27, 31, 34]. Three were rated ‘Fair’[26, 27, 31] and 1 rated ‘Good’[34]. Results are detailed in Table 4. Crowell[26] found riding a golf cart required least oxygen uptake per minute, then pulling clubs, and carrying clubs required the most oxygen uptake per minute. The study also noted golfers of lower handicaps (≤10) required less oxygen per minute when pulling or carrying clubs than golfers with higher handicaps (≥11). Dear et al.’s[27] value of 9.9 ± 1.7ml·kg^{-1}·min^{-1} equates to 34.4 ± 9.1% oxygen uptake reserve. Unverdorben et al.[34] found cardiac patients had a significantly higher %VO_2max whilst playing golf compared to healthy controls.

**Study**	**Quality Assessment**	**No. of Holes**	**Club Transportation**	**Oxygen Uptake (l·min^{-1})** *(mean ± SD)*	**Oxygen Uptake (ml·kg^{-1}·min^{-1})** *(mean ± SD)*	**%VO2max**
Crowell[26]	Fair	9<br>9<br>9	Riding a golf cart<br>Pulling clubs<br>Carrying clubs	1.05 ± 0.11<br>1.37 ± 0.03<br>1.50 ± 0.11	8.5<br>9.1<br>9.7	-
Dear et al. [27]	Fair	9	Pulling clubs	-	9.9 ± 1.7	-
Loy[31]	Fair	18	Carrying clubs	1.23 ± 0.11	-	
Unverdorben et al.[34]	Good	18<br><br>18	Pulling clubs	-	-	76.0 ± 13.1 *(cardiac patients)*<br>55.3 ± 9.1 *(controls)*

Table 4. Oxygen uptake during a round of golf.

Please refer to Appendix 6 for characteristics of the above studies.

Steps Taken

Three articles were found with steps taken as an outcome[29, 33, 38]. All studies rated ‘Fair’ in quality assessment. The included studies all involved an 18-hole round of golf. Studies found 11245±1351[29], 11948±1781[38], 16080±1195 (male)[33] and 16667±992 (female)[33] steps were taken during a round of golf. One study[29] found significant negative correlation between number of steps taken and: weight of the golf bag ($p<0.05$), energy expenditure ($p<0.01$) and minimum heart rate ($p<0.01$) of participants.

Distance Covered

Five studies detailed distance covered as an outcome[26, 27, 29, 32, 33]. Four of the studies were rated ‘Fair’[26, 27, 29, 33] and 1 study[33] was rated ‘Good’. Results are detailed in Table 5. With the exception of Crowell[26], all studies estimated between 8.7 and 11.25km walked for an 18-hole course, and 4.4 and 5.32km for a 9-hole

For peer review only - http://bmjopen.bmj.com/site/about/guidelines.xhtml

course. Distance covered is highly dependent on the individual golf course length. The course in Crowell's study is poorly described, but this may account for the shorter distance. A much shorter distance (3.18km) is walked riding a golf cart compared to pulling a golf cart or carrying clubs[26]. There is no notable difference in distance walked when pulling a golf cart compared to carrying clubs. Males walked longer distances than females. Zunzer et al.[32] noted a significant difference between male and female distance walked over 18 holes. However, in both studies[32, 33] and as is usual on golf courses, the men's course is longer than the women's. Tangen et al.[33] found that, when course length is accounted for, women (2.13 times the course length) walked significantly longer than men (1.98 times the course length).

Study	Quality Assessment	No. of holes	Club Transportation	Sex	Distance (km, mean ± SD)
Crowell[26]	Fair	18	Riding a golf cart<br>Pull cart<br>Carrying clubs	Male	3.18 ± 0.56*<br>7.37 ± 0.71*<br>6.47 ± 0.84*
Dear et al.[27]	Fair	9	Pull cart	Male	4.4 ± 3.6
Gabellieri[29]	Fair	18	Carrying clubs	Male	8.7 ± 0.6*
Zunzer et al.[32]	Good	18<br><br>9	Mixed<br>Mixed<br>Mixed<br>Mixed	Male<br>Female<br>Male<br>Female	10.54 ± 0.94<br>9.89 ± 0.81<br>5.32 ± 0.48<br>5.25 ± 0.76
Tangen et al.[33]	Fair	18	Mixed<br>Mixed	Male<br>Female	11.25 ± 0.83<br>10.00 ± 0.56

*converted to kilometres

Table 5. Distance covered in a round of golf.

Please refer to Appendix 6 for characteristics of the above studies.

Strength

One study listed strength as an outcome[39] and rated 'Good'. Sell et al.[39] found golfers with a lower handicap (<0) had significantly greater strength over a range of measures when compared to handicaps of 0-9 and 10-20. Tables are not listed for strength, flexibility or balance outcomes due to the heterogeneity of measurements.

Flexibility

One study listed flexibility as an outcome[39] and rated 'Good'. Sell et al.[39] found golfers with a lower handicap (<0) had significantly greater range of motion in several measures of shoulder, hip, torso flexibility than golfers with higher handicaps (0-9 and 10-20).

Balance

Five studies listed balance as an outcome[39-43]. Three studies rated 'Good'[39, 40, 42], two studies rated 'Fair'[41, 43]. Three studies focussed on older golfers[40-42] and all papers found elderly golfers had significantly better balance control when compared

For peer review only - http://bmjopen.bmj.com/site/about/guidelines.xhtml

to controls over a variety of measures. Tsang et al.[41] noted that the balance of elderly golfers was comparable to that of young controls (no significant difference).

Sell et al.[39] found golfers with better handicaps (<0) had significantly better single-leg balance than golfers with handicaps 0-9 and 10-20. Schachten et al.[43] noted a significant improvement in stroke patients after participating in a 10 week, 20 session golf putting intervention. However, a significant improvement was also noted in the comparator group and no significant difference was observed between groups.

DISCUSSION

Energy expenditure for an 18-hole round of golf appears to achieve the America College of Sports Medicine's (ACSM) recommendation of $1000kcal \cdot wk^{-1}$ [44], and could be separated into two 9-hole rounds. The length of time a round of golf takes can compensate for the low energy expenditure per minute. 50% of MET values stated are within the range of moderate intensity (3-5.9)[44]. Values for $\%HR_{max}$ are within light intensity (50-63%) and moderate intensity (64-76%)[14]. Using the mean age of golfer in UK (63yrs[45]), the mean range for moderate intensity is 101-119bpm – the large majority of data fall into this category.

There were varied results in oxygen uptake. In terms of VO_2max, studies classified golf as light (37-45% VO_2max), moderate (46-63% VO_2max) and vigorous (64-90%VO_2max)[44]. Many studies were close to, but did not reach, the moderate intensity threshold of $10.5\text{-}20.7ml \cdot kg^{-1} \cdot min^{-1}$ (3-5.9 METs), and would therefore be classified as light activity ($<10.5ml \cdot kg^{-1} \cdot min^{-1}$).

All included studies, on average, attained the often cited 10,000 steps[44] during an 18-hole round and, according to Tudor-Locke et al.[46], would be classed as moderate-to-vigorous physical activity. Distance walked is highly variable depending on the course; values range from 6.4-11.3km for an 18-hole round and 4.4-5.3km for a 9-hole round. In relation to strength, flexibility and balance, greater strength and range of motion was found in those with higher proficiency[39]. It is unclear whether this is due to increased volume of play, additional strength/flexibility work or whether these characteristics are likely to lead to a lower handicap. Furthermore, there appears to be better balance control in golfers. The complex motion while swinging a club and/or walking on uneven grounds during golf play may lead to improved stability, however this cannot be proven due to the methods employed in this study.

Evidence suggests use of an electric golf cart significantly reduces physical activity attained in terms of energy expenditure, heart rate and distance covered. Males expend more energy and walk further distances than females. However, it is likely that this due to greater body mass and course length played by males. When course length is accounted for, women walk significantly longer[33]. Skill level does not appear to affect physical activity accrued, with the possible exception of strength, balance and flexibility. The evidence is unclear whether course profile and age affect physical activity accrued.

This study is, to our knowledge, the first systematically conducted review to focus exclusively on golf and physical activity. It provides a general overview of physical activity accrued whilst playing golf. A rapid review was conducted due to time

For peer review only - http://bmjopen.bmj.com/site/about/guidelines.xhtml

constraints. Rapid reviews make use of streamlined methods and, due to this, are not subject to the same rigor as systematic reviews. For some outcomes, there was little available evidence. Furthermore, the sample sizes of included studies were generally small and ranged from 6 – 257 (median 22).

In agreement with the recent scoping review[10] and the Compendium of Physical Activities[11], golf can provide moderate intensity physical activity. Exercise intensity varies during the game itself. For certain populations, it may be primarily a low intensity physical activity. Shortfalls in intensity, however, are compensated for by the length of the game. Therefore, golf is a viable sport by which to achieve the physical activity recommendations[1]. Golfers may find it difficult to play enough during a week in order to reach PA recommendations, and may wish to supplement golf with another physical activity. Clinicians and policymakers can be encouraged to suggest golf as a form of physical activity in order to meet recommended levels and attain health benefits.

Further research is warranted to investigate whether strength and flexibility is accrued whilst playing golf as well as research examining the effect of modifiers such as age, course profile, disease characteristics and carrying or pulling clubs, on physical activity attained.

CONCLUSION

This rapid review identified 19 articles that examined golf and physical activity. Golf is primarily a moderate intensity physical activity, but may be low intensity or even high intensity depending on the population and various modifiers present. If able, golfers should walk the course, rather than ride a golf cart to maximise health benefits. Course profile, skill level and age may affect the amount of physical activity accrued, further research is required.

DECLARATION OF COMPETING INTERESTS

Although not for this project, AM has previously received funding to complete research from the World Golf Foundation. The World Golf Foundation committed to publishing whether results were positive, negative or equivocal, and had no influence on the conduct of this, or previous research.

FUNDING STATEMENT

This work was supported by the Medical Research Council (MRC; MR/K023209/1).

AUTHORS' CONTRIBUTIONS

Jack Luscombe: Lead researcher. Performed study selection, data extraction, quality assessment and narrative synthesis.

Dr Andrew Murray: Primary supervisor. Provided advice on methods including study selection criteria, data extraction and presentation of data. Performed phase one of the search strategy (previously published).

Evan Jenkins: Independently reviewed 10% of paper for study selection, data extraction and quality assessment.

For peer review only - http://bmjopen.bmj.com/site/about/guidelines.xhtml

Dr Daryll Archibald: Senior researcher. Performed phase two of search strategy and phase two study selection by title and abstract. Provided advice on methods including study selection criteria, data extraction and quality assessment.

DATA SHARING STATEMENT

There is no additional unpublished data.

REFERENCES

1. Department of Health PA, Health Improvement and Protection. Start active, stay active: A report on physical activity from the four home countries' Chief Medical Officers. 2011.
2. Reid H, Foster C. Infographic. Physical activity benefits for adults and older adults. British journal of sports medicine. 2016.
3. Warburton DE, Charlesworth S, Ivey A, Nettlefold L, Bredin SS. A systematic review of the evidence for Canada's Physical Activity Guidelines for Adults. The international journal of behavioral nutrition and physical activity. 2010;7:39.
4. O'Donovan G, Blazevich AJ, Boreham C, Cooper AR, Crank H, Ekelund U, et al. The ABC of Physical Activity for Health: a consensus statement from the British Association of Sport and Exercise Sciences. Journal of sports sciences. 2010;28(6):573-91.
5. Campbell-Jack D, Hinchliffe S, Rutherford L, Brown L, Gray L, Hovald P, et al. The Scottish Health Survey 2015. Edinburgh: Scottish Government; 2015.
6. Townsend N, Wickramasinghe K, Williams J, Bhatnagar P, Rayner M, Nuffield Department of Population Health. British Heart Foundation - Physical Activity Statistics. 2015.
7. Farrally MR, Cochran AJ, Crews DJ, Hurdzan MJ, Price RJ, Snow JT, et al. Golf science research at the beginning of the twenty-first century. Journal of sports sciences. 2003;21(9):753-65.
8. The Royal and Ancient. Golf around the world. The Royal and Ancient, 2015.
9. England Golf. England Golf Membership Survey 2014. 2014.
10. Murray AD, Daines L, Archibald D, Hawkes RA, Schiphorst C, Kelly P, et al. The relationships between golf and health: a scoping review. British journal of sports medicine. 2017;51(1):12-9.
11. Ainsworth BE, Haskell WL, Herrmann SD, Meckes N, Bassett DR, Jr., Tudor-Locke C, et al. 2011 Compendium of Physical Activities: a second update of codes and MET values. Medicine and science in sports and exercise. 2011;43(8):1575-81.
12. Haskell WL, Lee IM, Pate RR, Powell KE, Blair SN, Franklin BA, et al. Physical activity and public health: updated recommendation for adults from the American College of Sports Medicine and the American Heart Association. Medicine and science in sports and exercise. 2007;39(8):1423-34.
13. National Centre for Social Research. Health Survey for England. 2012.
14. American College of Sports Medicine., Pescatella LS, Arena R, Riebe D, Thompson PD. ACSM's guidelines for exercise testing and prescription. Ninth edition. ed. Baltimore: Lippincott Williams & Wilkins; 2013.
15. Murray AD, Daines L, Archibald D, Hawkes RA, Schiphorst C, Kelly P, et al. Infographic. Golf and health. British journal of sports medicine. 2017;51(1):20-1.
16. Peters M GC, McInerney P, Soares CB, Khalil H, Parker D. The Joanna Briggs Institute Reviewers' Manual 2015: Methodology for JBI Scoping Reviews. : The Joanna Briggs Institute; 2015.
17. Luscombe J, Murray A, Jenkins E, Archibald D. A rapid review to identify physical activity accrued whilst playing golf: PROSPERO 2017:CRD42017058237; [Available from: http://www.crd.york.ac.uk/PROSPERO/display_record.asp?ID=CRD42017058237.
18. Liberati A, Altman DG, Tetzlaff J, Mulrow C, Gotzsche PC, Ioannidis JP, et al. The PRISMA statement for reporting systematic reviews and meta-analyses of studies that evaluate healthcare interventions: explanation and elaboration. Bmj. 2009;339:b2700.

For peer review only - http://bmjopen.bmj.com/site/about/guidelines.xhtml

19. Watt A, Cameron A, Sturm L, Lathlean T, Babidge W, Blamey S, et al. Rapid versus full systematic reviews: validity in clinical practice? ANZ journal of surgery. 2008;78(11):1037-40.
20. Harker J, Kleijnen J. What is a rapid review? A methodological exploration of rapid reviews in Health Technology Assessments. Int J Evid Based Healthc. 2012;10.
21. Haby MM, Chapman E, Clark R, Barreto J, Reveiz L, Lavis JN. What are the best methodologies for rapid reviews of the research evidence for evidence-informed decision making in health policy and practice: a rapid review. Health Research Policy and Systems. 2016;14(1):83.
22. Tricco AC, Antony J, Zarin W, Strifler L, Ghassemi M, Ivory J, et al. A scoping review of rapid review methods. BMC Med. 2015;13:224.
23. Effective Public Health Practice Project. Quality Assessment Tool for Quantitative Studies 2004 [Available from: http://www.ephpp.ca/PDF/Quality%20Assessment%20Tool_2010_2.pdf.
24. National Heart Lung and Blood Insitute. Quality Assessment Tool for Observational Cohort and Cross-Sectional Studies 2014 [Available from: https://www.nhlbi.nih.gov/health-pro/guidelines/in-develop/cardiovascular-risk-reduction/tools/cohort.
25. Burkett LN, von Heijne-Fisher U. Heart rate and calorie expenditure of golfers carrying their clubs and walking flat and hilly golf courses. International Sports Journal. 1998;2(2):78-85.
26. Crowell BG. Energy cost of participation in golf as determined by telemetry: Oklahoma State University; 1970.
27. Dear JB, Porter MM, Ready AE. Energy expenditure during golfing and lawn mowing in older adult men. J Aging Phys Act. 2010;18(2):185-200.
28. Dobrosielski DA, Brubaker PH, Berry MJ, Ayabe M, Miller HS. The metabolic demand of golf in patients with heart disease and in healthy adults. J Cardiopulm Rehabil. 2002;22(2):96-104.
29. Gabellieri JM. The physiological demands of walking during golf: University of Rhode Island; 2011.
30. Lampley JH, Lampley PM, Howley ET. Caloric cost of playing golf. Res Q. 1977;48(3):637-9.
31. Loy SF. The effect of the game of golf on cardiopulmonary fitness of middle-aged men: California State University, Northridge; 1979.
32. Zunzer SC, von Duvillard SP, Tschakert G, Mangus B, Hofmann P. Energy expenditure and sex differences of golf playing. Journal of sports sciences. 2013;31(10):1045-53.
33. Tangen JO, Sunde A, Sageie J, Hagen PC, Kristoffersen B, Istad R, et al. In accordance with governmental recommendations—a study of golf and health. Journal of sports sciences. 2013;1:15-25.
34. Unverdorben M, Kolb M, Bauer I, Bauer U, Brune M, Benes K, et al. Cardiovascular load of competitive golf in cardiac patients and healthy controls. Medicine and science in sports and exercise. 2000;32(10):1674-8.
35. Broman G, Johnsson L, Kaijser L. Golf: a high intensity interval activity for elderly men. Aging clinical and experimental research. 2004;16(5):375-81.
36. Kras J, Larsen B. A comparison of the health benefits of walking and riding during a round of golf. International Sports Journal. 2002;6(1):112-6.
37. Stauch M, Liu Y, Giesler M, Lehmann M. Physical activity level during a round of golf on a hilly course. The Journal of sports medicine and physical fitness. 1999;39(4):321-7.
38. Kobriger SL, Smith J, Hollman JH, Smith AM. The contribution of golf to daily physical activity recommendations: how many steps does it take to complete a round of golf? Mayo Clin Proc. 2006;81(8):1041-3.
39. Sell TC, Tsai YS, Smoliga JM, Myers JB, Lephart SM. Strength, flexibility, and balance characteristics of highly proficient golfers. J Strength Cond Res. 2007;21(4):1166-71.
40. Gao KL, Hui-Chan CW, Tsang WW. Golfers have better balance control and confidence than healthy controls. Eur J Appl Physiol. 2011;111(11):2805-12.
41. Tsang WW, Hui-Chan CW. Effects of exercise on joint sense and balance in elderly men: Tai Chi versus golf. Medicine and science in sports and exercise. 2004;36(4):658-67.

For peer review only - http://bmjopen.bmj.com/site/about/guidelines.xhtml

42. Tsang WW, Hui-Chan CW. Static and dynamic balance control in older golfers. J Aging Phys Act. 2010;18(1):1-13.

43. Schachten T, Jansen P. The effects of golf training in patients with stroke: a pilot study. Int Psychogeriatr. 2015;27(5):865-73.

44. Garber CE, Blissmer B, Deschenes MR, Franklin BA, Lamonte MJ, Lee IM, et al. American College of Sports Medicine position stand. Quantity and quality of exercise for developing and maintaining cardiorespiratory, musculoskeletal, and neuromotor fitness in apparently healthy adults: guidance for prescribing exercise. Medicine and science in sports and exercise. 2011;43(7):1334-59.

45. Sports Marketing Surveys Inc. 2014 Golf Participation. 2014.

46. Tudor-Locke C, Craig CL, Brown WJ, Clemes SA, De Cocker K, Giles-Corti B, et al. How many steps/day are enough? For adults. The international journal of behavioral nutrition and physical activity. 2011;8:79.

FIGURE LEGENDS

Figure 1. Results of systematic electronic search.

For peer review only

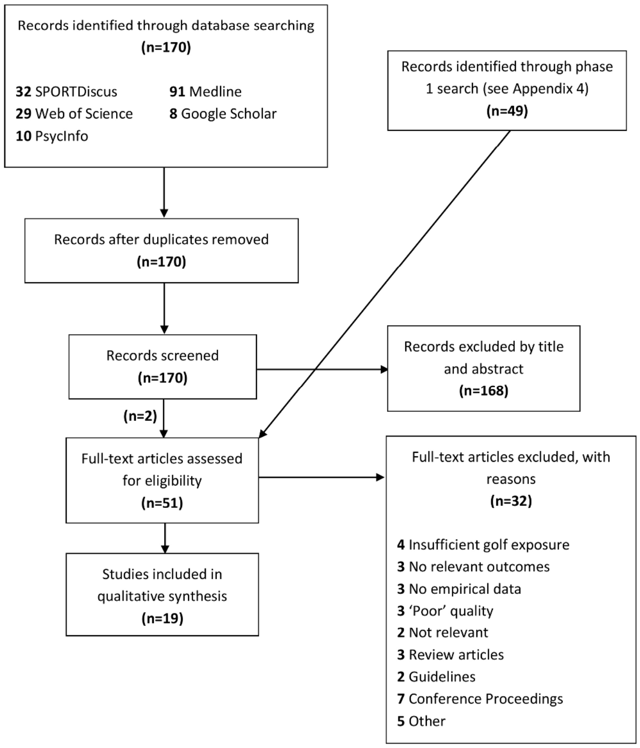

Figure 1. Results of systematic electronic search.

210x297mm (300 x 300 DPI)

Appendix 1. Searching protocol, phase 1

Step 1

SPORT Discus
1st search: from 1900, all articles, all languages
Search for: Golf AND health
Hits: 1364

2nd search
Search for: Golf AND health AND review
Hits: 56, of which 11 relevant studies identified

Google Advanced
1st search: Golf AND health AND review
Hits:487,000
First 200 articles reviewed for relevance, 15 appeared relevant

ProQuest dissertations
1st search: Golf AND health
Hits: 4, of which 2 relevant covering narrow aspect of topic.

Reference section of useful studies reviewed.

Step 2

The main category – terms are:

1. Golf
   Plus/minus
2. Health OR illness OR injur* OR fitness OR mortality OR morbidity

SPORTDiscus

1st search: from 1900, all articles, all languages
Search for: Golf AND health OR illness OR injur* OR fitness OR mortality OR morbidity
Hits: 3781

2nd search: from 1900, all articles excluding magazine, all languages
Search for: Golf AND health OR illness OR injur* OR fitness OR mortality OR morbidity excluding magazines
Hits: 744

Web of Science

1st search: from 1900, all articles, all languages,
Search for: Golf AND health OR illness OR injur* OR fitness OR mortality OR morbidity

For peer review only

Hits: 559

PsycINFO

1st search from 1900, all articles, all languages
Search for: Golf
Topics Hits: 832

Medline

1st search: from 1900, all articles, all languages
Search for: Golf
Hits: 1721

Google scholar

1st search: from 1900, articles and patents, include citations
Search for: Golf AND health OR illness OR injury OR fitness OR mortality OR morbidity
Hits: 154000, >too many

2nd Search: from 1900, articles and patents, include citations
Search for: Golf AND health OR illness OR injury OR fitness OR mortality OR morbidity
Within title
Hits: 185

Initial database search

Hits: 4041 before duplicates
3167 once duplicates removed

Grey Literature

Google (advanced search)

British Journal of Sports Medicine Domain
1st search: from 1900, all articles, all languages
Search for: Golf AND health OR illness OR injury OR fitness OR mortality OR morbidity AND specify URL http://bjsm.bmj.com/
Hits: 548
https://www.google.co.uk/search?as_q=golf&as_epq=&as_oq=health+illness+injury++fitness++mortality+morbidity+&as_eq=&as_nlo=&as_nhi=&lr=&cr=&as_qdr=all&as_sitesearch=http://bjsm.bmj.com/&as_occt=any&safe=images&as_filetype=&as_right=&gws_rd=cr&ei=Eq1AVue-OsfTU6XPtOAC#q=golf+health+OR+illness+OR+injury+OR+fitness+OR+mortality+O R+morbidity+site:http://bjsm.bmj.com/&as_qdr=all&start=20

For peer review only

World Golf Foundation
1st search: from 1900, all articles, all languages
Search for: Golf AND health OR illness OR injury OR fitness OR mortality OR morbidity AND specify URL http://www.worldgolffoundation.org/
Hits:11
https://www.google.com/search?as_q=golf&as_epq=&as_oq=health+illness+injury+fitness+ mortality+morbidity+&as_eq=&as_nlo=&as_nhi=&lr=&cr=&as_qdr=all&as_sitesearch=http %3A%2F%2Fwww.worldgolffoundation.org%2F&as_occt=any&safe=images&as_filetype= &as_rights=

Royal and Ancient
1st search: from 1900, all articles, all languages
Search for: Golf AND health OR illness OR injury OR fitness OR mortality OR morbidity AND specify URL http://www.randa.org/
Hits: 133
https://www.google.com/search?as_q=golf&as_epq=&as_oq=health+illness+injury+fitness+ mortality+morbidity+&as_eq=&as_nlo=&as_nhi=&lr=&cr=&as_qdr=all&as_sitesearch=http %3A%2F%2Fwww.randa.org%2F&as_occt=any&safe=images&as_filetype=&as_rights=

Faculty of Sports and Exercise Medicine
1st search: from 1900, all articles, all languages
Search for: Golf AND health OR illness OR injury OR fitness OR mortality OR morbidity AND specify URL http://www.fsem.ac.uk/
Hits: 8
https://www.google.com/search?as_q=golf&as_epq=&as_oq=health+injury+illness+morbidit y+mortality&as_eq=&as_nlo=&as_nhi=&lr=&cr=&as_qdr=all&as_sitesearch=http%3A%2F %2Fwww.fsem.ac.uk%2F&as_occt=any&safe=images&as_filetype=&as_rights=

American College for Sports Medicine
1st search: from 1900, all articles, all languages
Search for: Golf AND health OR illness OR injury OR fitness OR mortality OR morbidity AND specify URL http://www.acsm.org/
Hits: 26
https://www.google.com/search?as_q=golf&as_epq=&as_oq=health+injury+illness+morbidit y+mortality&as_eq=&as_nlo=&as_nhi=&lr=&cr=&as_qdr=all&as_sitesearch=http%3A%2F %2Fwww.acsm.org%2F&as_occt=any&safe=images&as_filetype=&as_rights=

ProQuest dissertations

1st search: from 1900, all articles, all languages
Search for: Golf AND health OR illness OR injury OR fitness OR mortality OR morbidity

Hits: 42740 >too many

2nd search: Golf AND Health OR illness OR injur* OR fitness OR mortality OR morbidity in title or abstract
Hits: 175 Duplicates 115

World Health Organisation- International Clinical Trials Registry Platform

1st search
Search for: Golf
Hits: 2
http://apps.who.int/trialsearch/Trial2.aspx?TrialID=NCT02544399 (relevant)
http://apps.who.int/trialsearch/Trial2.aspx?TrialID=EUCTR2005-003458-81-IT (not relevant)

Initial Grey Literature Databases search

Hits: 903

For peer review only

Appendix 2. Searching protocol, phase 2

SPORTDiscus
From 1900, all articles, all languages
Search for: Golf AND health
Hits: 32

Web of Science
From 1900, all articles, all languages,
Search for: Golf AND health
Hits: 29

PsycInfo
From 1900, all articles, all languages
Search for: Golf
Hits: 10

Medline
From 1900, all articles, all languages
Search for: Golf
Hits: 91

Google Scholar
From 1900, articles and patents, include citations
Search for: Golf AND health OR illness OR injury OR fitness OR mortality OR morbidity
Within title
Hits: 8

For peer review only

Appendix 3. Sample Data Extraction Form.

Bibliographic Information

Study ID:	Date of extraction:	Extracted by:	Checked by:
Year published:	Country:		
Title:			
Author(s):			

Study Characteristics

Aims/purpose:
Study design:
Analytical methods deployed:
Study procedures:

Participants Characteristics

Age:	Gender:	Sample size:
Any disease characteristics:	Handicap/Average score:	

Intervention

Intervention Type:
Comparator:
Setting:

For peer review only

**Duration:**	
**Modifiers:**	
Sex	
Hills	
Golf Carts/Pulling/Carrying Clubs	
Age	
Other	

For peer review only

Outcomes

**METS**	
**Energy expenditure**	
**O_2 intake**	
**Heart rate**	
**Steps taken**	
**Distance covered**	
**Strength**	
**Flexibility**	
**Balance**	
**Sedentary Behaviour**	
**Outcome Measures:**	

Results

**Key findings:**
**Limitations:**

For peer review only

Appendix 4. Results of phase one of the systematic electronic search.

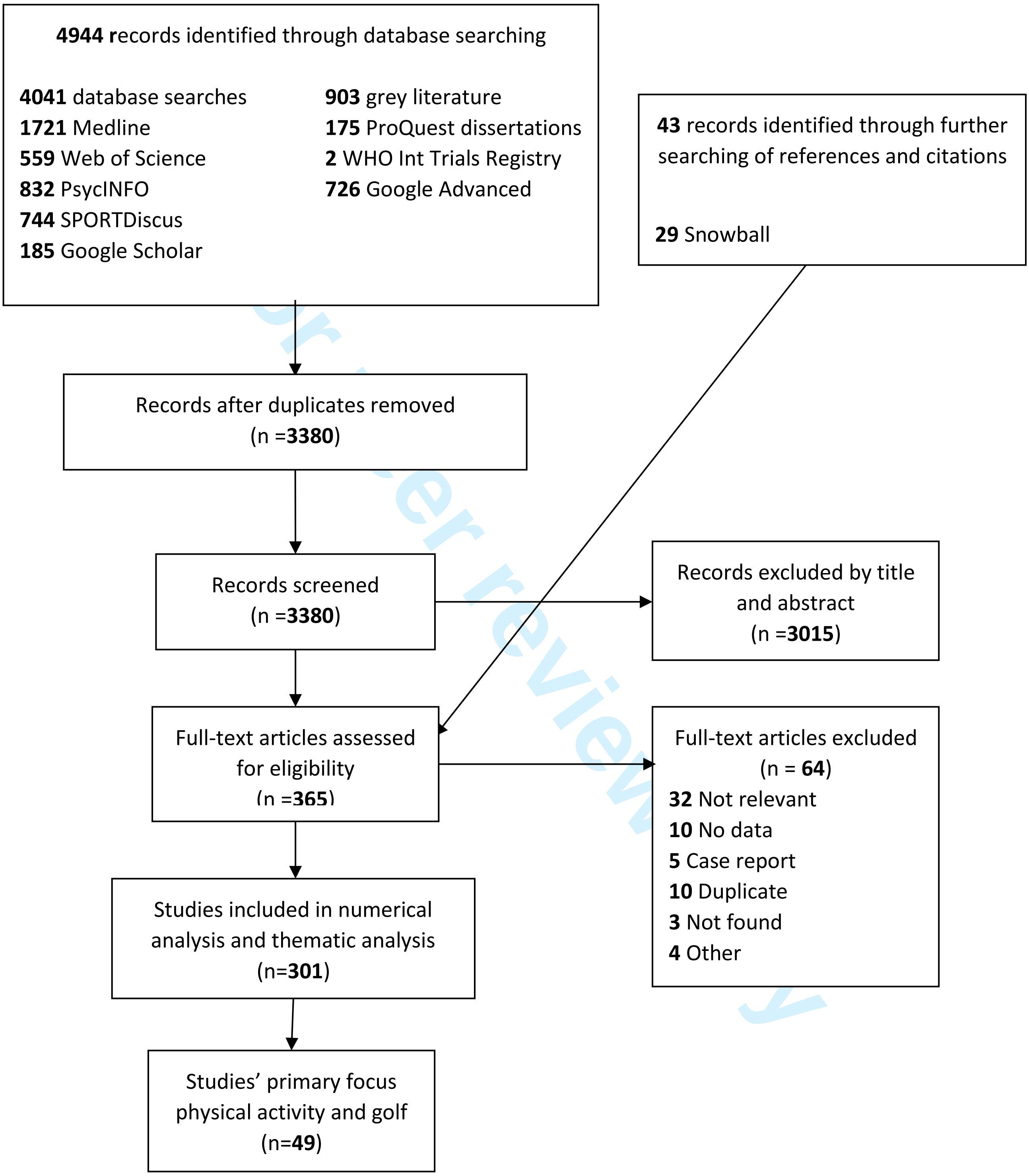

Adapted from Murray et al. [10]

Appendix 5. Citations of studies included in the rapid review

1. Burkett LN, von Heijne-Fisher U. Heart rate and calorie expenditure of golfers carrying their clubs and walking flat and hilly golf courses. International Sports Journal. 1998;2(2):78-85.

2. Broman G, Johnsson L, Kaijser L. Golf: a high intensity interval activity for elderly men. Aging clinical and experimental research. 2004;16(5):375-81.

3. Crowell BG. Energy cost of participation in golf as determined by telemetry: Oklahoma State University; 1970.

4. Dear JB, Porter MM, Ready AE. Energy expenditure during golfing and lawn mowing in older adult men. J Aging Phys Act. 2010;18(2):185-200.

5. Dobrosielski DA, Brubaker PH, Berry MJ, Ayabe M, Miller HS. The metabolic demand of golf in patients with heart disease and in healthy adults. J Cardiopulm Rehabil. 2002;22(2):96-104.

6. Gabellieri JM. The physiological demands of walking during golf: University of Rhode Island; 2011.

7. Gao KL, Hui-Chan CW, Tsang WW. Golfers have better balance control and confidence than healthy controls. Eur J Appl Physiol. 2011;111(11):2805-12.

8. Kobriger SL, Smith J, Hollman JH, Smith AM. The contribution of golf to daily physical activity recommendations: how many steps does it take to complete a round of golf? Mayo Clin Proc. 2006;81(8):1041-3.

9. Kras J, Larsen B. A comparison of the health benefits of walking and riding during a round of golf. International Sports Journal. 2002;6(1):112-6.

10. Lampley JH, Lampley PM, Howley ET. Caloric cost of playing golf. Res Q. 1977;48(3):637-9.

11. Loy SF. The effect of the game of golf on cardiopulmonary fitness of middle-aged men: California State University, Northridge; 1979.

12. Sell TC, Tsai YS, Smoliga JM, Myers JB, Lephart SM. Strength, flexibility, and balance characteristics of highly proficient golfers. J Strength Cond Res. 2007;21(4):1166-71.

13. Stauch M, Liu Y, Giesler M, Lehmann M. Physical activity level during a round of golf on a hilly course. The Journal of sports medicine and physical fitness. 1999;39(4):321-7.

14. Tsang WW, Hui-Chan CW. Effects of exercise on joint sense and balance in elderly men: Tai Chi versus golf. Medicine and science in sports and exercise. 2004;36(4):658-67.

15. Tsang WW, Hui-Chan CW. Static and dynamic balance control in older golfers. J Aging Phys Act. 2010;18(1):1-13.

For peer review only

16. Unverdorben M, Kolb M, Bauer I, Bauer U, Brune M, Benes K, et al. Cardiovascular load of competitive golf in cardiac patients and healthy controls. Medicine and science in sports and exercise. 2000;32(10):1674-8.

17. Zunzer SC, von Duvillard SP, Tschakert G, Mangus B, Hofmann P. Energy expenditure and sex differences of golf playing. Journal of sports sciences. 2013;31(10):1045-53.

18. Tangen JO, Sunde A, Sageie J, Hagen PC, Kristoffersen B, Istad R, et al. In accordance with governmental recommendations—a study of golf and health. Journal of sports sciences. 2013;1:15-25.

19. Schachten T, Jansen P. The effects of golf training in patients with stroke: a pilot study. Int Psychogeriatr. 2015;27(5):865-73.

For peer review only

Appendix 6. Characteristics of included studies

Study ID	Author, publication year	Country	Sample size	Participants' age (mean years)	Participants' gender (% Male)	Participants' handicap or average score (mean strokes)	Participant's disease characteristics	Duration	Modifiers	Relevant outcome measures
1	Burkett, 1998	USA	10	24.0	100	<80† (50%)<br>80-95† (50%)	Healthy	3 x 18 holes	Course profile; skill level	Energy expenditure; heart rate
3	Broman, 2004	Sweden	19	27.0 (31%)<br>50.0 (37%)<br>75.0 (31%)	100	NR	1 x Polymyalgia rheumatica; 7 x "back problems"; 2 x knee pain	18 holes	Age; areas of golf course	Heart rate
4	Crowell, 1970	USA	7	42.7	100	11.6	NR	3 x 9 holes	Club transportation; shot type	Energy expenditure; oxygen intake; heart rate; distance covered
7	Dear, 2010	USA	18	71.2	100	NR	Healthy	9 holes	-	METs; energy expenditure; oxygen intake; heart rate; distance covered
8	Dobrosielski, 2002	USA	20	63.6 (50%)<br>65.8 (50%)	100	NR	2 x myocardial infarction<br>4 x coronary artery bypass graft<br>2 x percutaneous coronary intervention<br>1 x cardiomyopathy	9 holes	Heart disease	METs; energy expenditure

11	Gabellieri, 2011	USA	13	28.5	100	97.1[†]	NR	18 holes	Weight	METs; energy expenditure; heart rate; steps taken; distance covered
12	Gao, 2011	China	23	66.2 (47.8%)<br>71.3 (52.2%)	100	NR	Healthy	NA	-	Balance
24	Kobriger, 2006	USA	42	55	28.6	NR	NR	3 x 18 holes	Sex	Steps taken
25	Kras, 2002	USA	12	42-57	100	7-21	NR	3 x 9 holes	Course profile, club transportation	Heart rate
26	Lampley, 1977	USA	22	34 (50%)<br>32 (50%)	50	19	NR	9 holes	Sex	Energy expenditure
27	Loy, 1979	USA	6	52.5	100	15.7	Healthy	18 holes	-	Energy expenditure; oxygen intake; heart rate
34	Sell, 2008	USA	257	45.5	100	<0 (17.5%)<br>0-9 (46.7%)<br>10-20 (35.8%)	NR	NA	Skill level	Strength; balance; flexibility
35	Stauch, 2003	USA	30	53	70	29	9 x cardiovascular disease	18 holes	Course profile; sex	Heart rate
37	Tsang, 2004	China	35	69.6 (34.3%)<br>66.2 (31.4%)<br>71.3 (34.3%)<br>20.3 (34.3%)	100	NR	NR	NA	-	Balance
38	Tsang, 2010	China	23	66.2 (47.8%)<br>71.3 (52.2%)	100	NR	NR	NA	-	Balance
39	Unverdor ben, 2000	Germany	28	65.3 (71.4%)<br>62.0 (28.6%)	100	NR	11 x coronary artery disease; 10 x post myocardial infarction; 2 x	18 holes	Heart disease	METs; oxygen intake; heart rate

For peer review only

							peripheral arterial occlusive disease; 14 x arrhythmias; 1 x post-myocarditis; 2 x valve disease; 1 x diabetes mellitus; 12 x hypertension			
41	Zunzer, 2013	Austria	66	53.3	63.6	26.4 (51.5%)<br>20.4 (48.5%)	NR	9 holes or 18 holes	Sex; course profile; club transportation; age	METs; energy expenditure; heart rate; distance covered
49	Tangen, 2013	Norway	29	44.2	48.3	27.3	NR	18 holes	Sex; course profile; age; skill level	METs; energy expenditure; heart rate; steps taken; distance covered
50	Schachen, 2015	Germany	14	55.1 (50%)<br>53.1 (50%)	NR	NR	Stroke	10 weeks (2 putting session/week)	-	Balance

NR: Not reported
NA: not applicable
**calculated*
†*average score*

For peer review only

Appendix 7. Quality assessment of included studies.

Author, year	Clear objective	Clearly defined study population	Participation rate of eligible persons >50%	Recruited from same or similar populations + inclusion and exclusion criteria prespecified	Sample size justification, power description, or variance and effect estimates	Exposures measured prior to outcome being measured	Sufficient timeframe	Different levels of exposure examined	Exposure measures clearly defined, valid, reliable and implemented consistently	Exposure assessed more than once over time	Outcome measures clearly defined, valid, reliable and implemented consistently	Outcome assessors blinded to exposure status	Loss to follow-up <20%	Key confounding variables measured and adjusted for	Overall Rating
Burkett, 1998	Y	N	NA	CD	N	NA	Y	Y	N	NA	Y	CD	NA	N	**Fair**
Broman, 2004	Y	Y	NA	Y	N	NA	Y	N	N	NA	Y	NA	NA	N	**Fair**
Crowell, 1970	Y	Y	NA	N	N	NA	Y	Y	Y	NA	Y	CD	NA	Y	**Fair**
Dear, 2010	Y	Y	NA	Y	N	NA	Y	N	N	NA	Y	CD	NA	N	**Fair**
Dobrosielski, 2002	Y	Y	NA	N	N	NA	Y	N	N	NA	Y	CD	NA	N	**Fair**
Gabellieri, 2011	Y	N	NA	CD	Y	NA	Y	N	Y	N	Y	NA	NA	N	**Fair**
Gao, 2011	Y	Y	NA	Y	Y	NA	Y	N	Y	NA	Y	CD	NA	Y	**Good**
Kobriger, 2006	Y	N	NA	Y	Y	NA	Y	Y	N	N	Y	NA	NA	N	**Fair**
Kras, 2002	Y	N	NA	CD	Y	NA	Y	Y	Y	N	Y	CD	NA	N	**Fair**
Lampley, 1977	Y	N	NA	CD	N	NA	Y	N	N	N	Y	NA	NA	Y	**Fair**
Loy, 1979	Y	Y	NA	N	N	NA	Y	N	N	N	Y	NA	NA	N	**Fair**
Sell, 2008	Y	N	NA	CD	N	Y	CD	Y	Y	NA	Y	CD	NA	N	**Good**
Stauch, 2003	Y	Y	NA	N	N	NA	Y	Y	Y	NA	Y	NA	NA	N	**Fair**
Tsang, 2004	Y	N	NA	N	N	Y	Y	N	Y	N	Y	CD	NA	Y	**Fair**

For peer review only

Tsang, 2010	Y	Y	NA	Y	N	Y	Y	N	Y	N	Y	CD	NA	Y	**Good**
Unverdorben, 2000	Y	Y	NA	N	N	NA	Y	N	Y	N	Y	CD	NA	Y	**Good**
Zunzer, 2013	Y	Y	NA	Y	N	NA	Y	Y	Y	N	Y	CD	NA	Y	**Good**
Tangen, 2013	Y	N	NA	CD	N	NA	Y	Y	N	N	Y	CD	NA	Y	**Fair**
Schachen, 2015	Y	N	NA	N	Y	NA	Y	N	Y	NA	Y	CD	NA	N	**Fair**

Y: yes
N: no
NA: not applicable
CD: can't determine
NR: not reported

For peer review only

PRISMA 2009 Checklist

Section/topic	#	Checklist item	Reported on page #
**TITLE**			
Title	1	Identify the report as a systematic review, meta-analysis, or both.	0
**ABSTRACT**			
Structured summary	2	Provide a structured summary including, as applicable: background; objectives; data sources; study eligibility criteria, participants, and interventions; study appraisal and synthesis methods; results; limitations; conclusions and implications of key findings; systematic review registration number.	1
**INTRODUCTION**			
Rationale	3	Describe the rationale for the review in the context of what is already known.	2
Objectives	4	Provide an explicit statement of questions being addressed with reference to participants, interventions, comparisons, outcomes, and study design (PICOS).	2
**METHODS**			
Protocol and registration	5	Indicate if a review protocol exists, if and where it can be accessed (e.g., Web address), and, if available, provide registration information including registration number.	2
Eligibility criteria	6	Specify study characteristics (e.g., PICOS, length of follow-up) and report characteristics (e.g., years considered, language, publication status) used as criteria for eligibility, giving rationale.	3-4
Information sources	7	Describe all information sources (e.g., databases with dates of coverage, contact with study authors to identify additional studies) in the search and date last searched.	3
Search	8	Present full electronic search strategy for at least one database, including any limits used, such that it could be repeated.	Appendices 1 + 2
Study selection	9	State the process for selecting studies (i.e., screening, eligibility, included in systematic review, and, if applicable, included in the meta-analysis).	3-4
Data collection process	10	Describe method of data extraction from reports (e.g., piloted forms, independently, in duplicate) and any processes for obtaining and confirming data from investigators.	4
Data items	11	List and define all variables for which data were sought (e.g., PICOS, funding sources) and any assumptions and simplifications made.	4
Risk of bias in individual studies	12	Describe methods used for assessing risk of bias of individual studies (including specification of whether this was done at the study or outcome level), and how this information is to be used in any data synthesis.	4
Summary measures	13	State the principal summary measures (e.g., risk ratio, difference in means).	4
Synthesis of results	14	Describe the methods of handling data and combining results of studies, if done, including measures of consistency (e.g., I^2) for each meta-analysis.	4

For peer review only - http://bmjopen.bmj.com/site/about/guidelines.xhtml

PRISMA 2009 Checklist

Section/topic	#	Checklist item	Reported on page #
Risk of bias across studies	15	Specify any assessment of risk of bias that may affect the cumulative evidence (e.g., publication bias, selective reporting within studies).	NA
Additional analyses	16	Describe methods of additional analyses (e.g., sensitivity or subgroup analyses, meta-regression), if done, indicating which were pre-specified.	NA
**RESULTS**			
Study selection	17	Give numbers of studies screened, assessed for eligibility, and included in the review, with reasons for exclusions at each stage, ideally with a flow diagram.	4-5
Study characteristics	18	For each study, present characteristics for which data were extracted (e.g., study size, PICOS, follow-up period) and provide the citations.	5-6
Risk of bias within studies	19	Present data on risk of bias of each study and, if available, any outcome level assessment (see item 12).	6
Results of individual studies	20	For all outcomes considered (benefits or harms), present, for each study: (a) simple summary data for each intervention group (b) effect estimates and confidence intervals, ideally with a forest plot.	6-13
Synthesis of results	21	Present results of each meta-analysis done, including confidence intervals and measures of consistency.	NA
Risk of bias across studies	22	Present results of any assessment of risk of bias across studies (see Item 15).	6
Additional analysis	23	Give results of additional analyses, if done (e.g., sensitivity or subgroup analyses, meta-regression [see Item 16]).	NA
**DISCUSSION**			
Summary of evidence	24	Summarize the main findings including the strength of evidence for each main outcome; consider their relevance to key groups (e.g., healthcare providers, users, and policy makers).	14-15
Limitations	25	Discuss limitations at study and outcome level (e.g., risk of bias), and at review-level (e.g., incomplete retrieval of identified research, reporting bias).	15
Conclusions	26	Provide a general interpretation of the results in the context of other evidence, and implications for future research.	15
**FUNDING**			
Funding	27	Describe sources of funding for the systematic review and other support (e.g., supply of data); role of funders for the systematic review.	15

From: Moher D, Liberati A, Tetzlaff J, Altman DG, The PRISMA Group (2009). Preferred Reporting Items for Systematic Reviews and Meta-Analyses: The PRISMA Statement. PLoS Med 6(7): e1000097. doi:10.1371/journal.pmed1000097

For more information, visit: www.prisma-statement.org.

For peer review only - http://bmjopen.bmj.com/site/about/guidelines.xhtml
